# Allele-specific quantitation of *ATXN3* and *HTT* transcripts in polyQ disease models

**DOI:** 10.1186/s12915-023-01515-3

**Published:** 2023-02-01

**Authors:** Paweł Joachimiak, Adam Ciesiołka, Emilia Kozłowska, Paweł M. Świtoński, Grzegorz Figura, Agata Ciołak, Grażyna Adamek, Magdalena Surdyka, Żaneta Kalinowska-Pośka, Maciej Figiel, Nicholas S. Caron, Michael R. Hayden, Agnieszka Fiszer

**Affiliations:** 1 Department of Medical Biotechnology, Institute of Bioorganic Chemistry, Polish Academy of Sciences, Noskowskiego 12/14, 61-704 Poznan, Poland; 2 Department of Molecular Neurobiology, Institute of Bioorganic Chemistry, Polish Academy of Sciences, Noskowskiego 12/14, 61-704 Poznan, Poland; 3 Centre for Molecular Medicine and Therapeutics, BC Children’s Hospital Research Institute, Department of Medical Genetics, University of British Columbia, Vancouver, BC V5Z 4H4 Canada

**Keywords:** Polyglutamine diseases, Huntington’s disease, Spinocerebellar ataxia type 3, ddPCR, Allele-specific quantitation, SNP

## Abstract

**Background:**

The majority of genes in the human genome is present in two copies but the expression levels of both alleles is not equal. Allelic imbalance is an aspect of gene expression relevant not only in the context of genetic variation, but also to understand the pathophysiology of genes implicated in genetic disorders, in particular, dominant genetic diseases where patients possess one normal and one mutant allele. Polyglutamine (polyQ) diseases are caused by the expansion of CAG trinucleotide tracts within specific genes. Spinocerebellar ataxia type 3 (SCA3) and Huntington’s disease (HD) patients harbor one normal and one mutant allele that differ in the length of CAG tracts. However, assessing the expression level of individual alleles is challenging due to the presence of abundant CAG repeats in the human transcriptome, which make difficult the design of allele-specific methods, as well as of therapeutic strategies to selectively engage CAG sequences in mutant transcripts.

**Results:**

To precisely quantify expression in an allele-specific manner, we used SNP variants that are linked to either normal or CAG expanded alleles of the ataxin-3 (*ATXN3*) and huntingtin (*HTT*) genes in selected patient-derived cell lines. We applied a SNP-based quantitative droplet digital PCR (ddPCR) protocol for precise determination of the levels of transcripts in cellular and mouse models. For HD, we showed that the process of cell differentiation can affect the ratio between endogenous alleles of *HTT* mRNA. Additionally, we reported changes in the absolute number of the *ATXN3* and *HTT* transcripts per cell during neuronal differentiation. We also implemented our assay to reliably monitor, in an allele-specific manner, the silencing efficiency of mRNA-targeting therapeutic approaches for HD. Finally, using the humanized Hu128/21 HD mouse model, we showed that the ratio of normal and mutant *HTT* transgene expression in brain slightly changes with the age of mice.

**Conclusions:**

Using allele-specific ddPCR assays, we observed differences in allele expression levels in the context of SCA3 and HD. Our allele-selective approach is a reliable and quantitative method to analyze low abundant transcripts and is performed with high accuracy and reproducibility. Therefore, the use of this approach can significantly improve understanding of allele-related mechanisms, e.g., related with mRNA processing that may be affected in polyQ diseases.

**Supplementary Information:**

The online version contains supplementary material available at 10.1186/s12915-023-01515-3.

## Background

Polyglutamine (polyQ) diseases are a family of neurodegenerative disorders that include spinocerebellar ataxia type 3 (SCA3) and Huntington’s disease (HD), caused by a CAG repeat expansion in the coding region of specific genes [1, 2]. Due to an autosomal dominant inheritance pattern, most of polyQ disease patients carry a normal (wild type, WT) and mutant (MUT) allele. Normal alleles usually contain 5–30 CAG repeats, while mutant alleles, encoding proteins with long stretches of glutamines, are characterized by more than 39 CAG repeats in HD and above 60 CAG repeats in the case of SCA3 [2, 3].

SCA3 is caused by a CAG repeat expansion in the exon 10 of the ataxin-3 (*ATXN3*) gene and typically manifests with dysfunction and degeneration of neurons in cerebellum and spinocerebellar tracts [4]. HD, in turn, is caused by a CAG repeat expansion in the first exon of the huntingtin (*HTT*) gene. In HD, the mutation leads mostly to degeneration of striatal and cortical neurons. However, other brain regions may also be substantially affected in both SCA3 and HD [5].

A mutant protein is generally recognized to be the main pathogenic factor in polyQ diseases. Expanded polyQ tracts induce the formation of misfolded protein aggregates that disturb cellular homeostasis and lead to neuronal death [2, 6–9]. The exact role of mutant transcripts in pathogenic mechanisms in polyQ diseases is being unraveled [10–13]. RNA-related studies have demonstrated so far: incomplete splicing of *HTT* mRNA [14], abnormal interactions of RNAs containing expanded CAG repeats with proteins [15, 16], and RNA foci formation [17, 18], and potential importance of alternative polyadenylation of various polyQ transcripts [19]. Furthermore, mutant polyQ disease transcripts have been shown to be promising therapeutic targets in strategies that aim to downregulate mutant gene expression [2, 9, 20].

Molecular examination of endogenous polyQ transcripts is a challenging task. PolyQ disease genes are expressed at low levels across all tissues, including the brain regions that are mostly affected by the disease [21]. Any method that seeks to inspect polyQ disease transcripts needs to be highly accurate and sensitive. Moreover, if method of choice requires discrimination between WT and MUT alleles, the CAG sequence region cannot be used to design a primer or probe. One possible solution to overcome these limitations is to utilize heterozygous SNP variants present in transcripts. Droplet digital PCR (ddPCR) utilizes droplet-based microfluidics and compartmentalization to provide absolute quantification of analyzed molecules and has been used for allele-selective quantification of transcripts [22–25].

In this study, we first identified heterozygous SNP variants in *ATXN3* and *HTT* cDNAs from patient-derived cell lines. Next, we adapted ddPCR to perform accurate and quantitative determination of the WT/MUT allele ratio and estimation of the number of the *ATXN3* and *HTT* transcripts per cell. We performed our analyses on fibroblasts and induced pluripotent stem cells (iPSCs), as well as neural stem cells (NSCs) and neurons, differentiated from iPSCs. Using these cell lines, we demonstrated changes in WT/MUT allele expression ratios emerging from a neuronal differentiation process. Additionally, we determined the number of *HTT* WT and MUT transgene copies and their expression ratio in the brain tissue of biallelic HD mice. The obtained results provide a starting point for further investigation of the relevance of expression of WT and MUT alleles to the pathology of polyQ diseases.

## Results

### Examination of endogenous ATXN3 transcripts

**Fig. 1:**
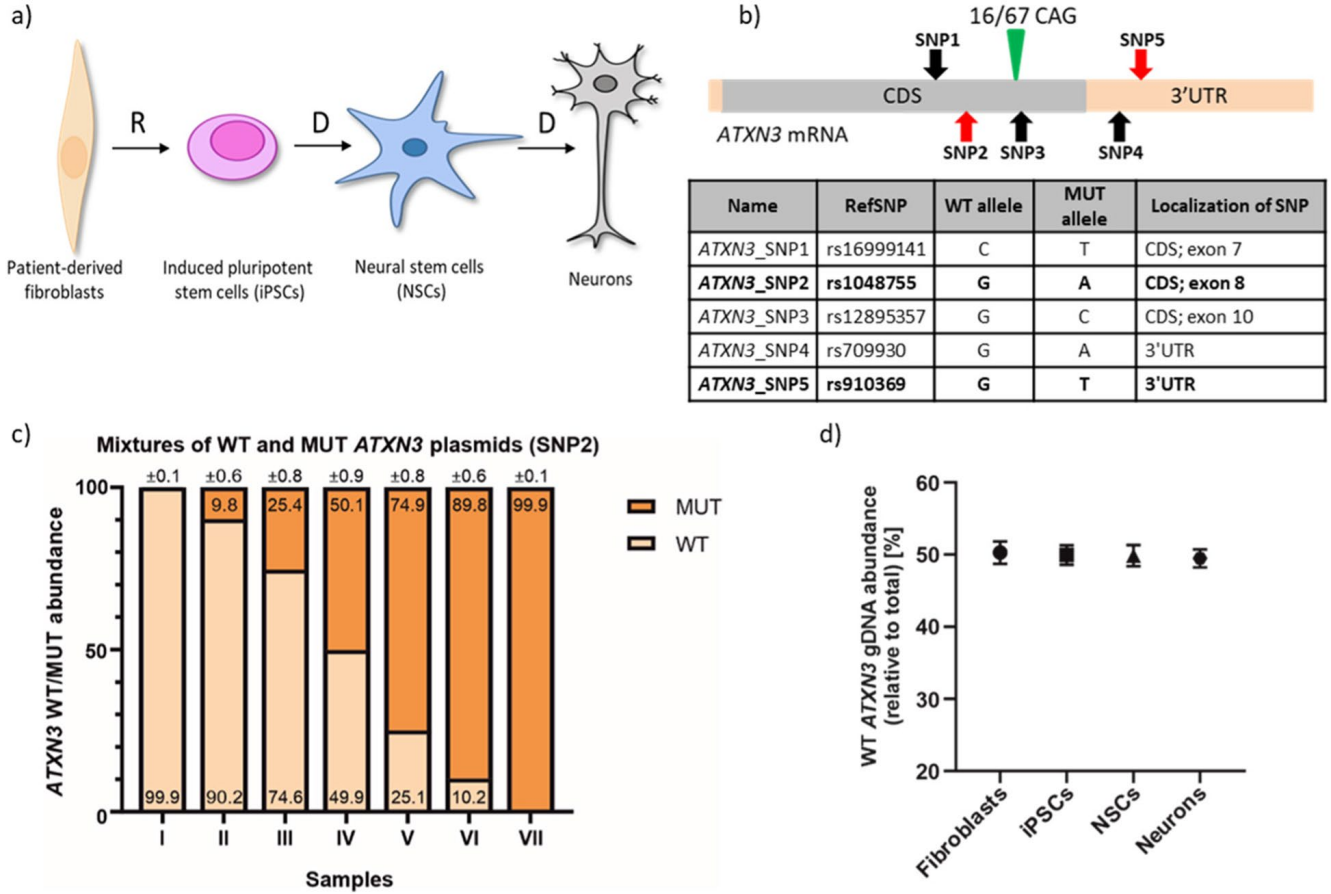
SNP-based allele-specific ddPCR assays for ATXN3 transcript. ***a*** SCA3 cell lines used; R—reprogramming; D—differentiation. ***b*** Scheme of ATXN3 transcript with marked CAG repeat tract (green triangle) and SNP variants identified in SCA3 cell lines (red arrows*—*SNPs used in ddPCR assays; black arrows*—*other identified SNPs); light orange box*—*UTRs. Table presents all SNP variants identified in analyzed SCA3 cell lines. Bolded SNPs were selected to be targets for ddPCR assays, and they will be hereinafter referred to as ATXN3_SNP2 and ATXN3_SNP5 in the text. CDS—coding sequence. RefSNP number according to the Single Nucleotide Polymorphism Database (dbSNP). ***c*** Results from ddPCR analysis of ATXN3_SNP2 assay specificity performed on seven samples with predefined ratios of WT/MUT ATXN3 plasmids (samples: I*—*100% WT and 0% MUT; II*—*90% WT and 10% MUT; III*—*75% WT and 25% MUT; IV*—*50% WT and 50% MUT; V*—*25% WT and 75% MUT; VI*—*10% WT and 90% MUT; VII*—*0% WT and 100% MUT). Precise values are indicated on WT/MUT bars ± poisson error. ***d*** Percentage ratio of ATXN3 WT allele obtained with ddPCR ATXN3_SNP5 assay using gDNA from indicated SCA3 cell lines. Three biological replicates were performed. These data are presented as means ± SD

For the ddPCR assay measuring the expression of *ATXN3* WT and MUT alleles, we used a set of patient-derived cell lines with the SCA3 genetic background: fibroblasts, iPSCs, NSCs, and neurons (characterized by 16/67 CAG repeats in *ATXN3*) (Fig. 1a).

#### Identification of SNP variants in ATXN3 transcripts

To identify SNP variants, we cloned and sequenced *ATXN3* cDNA from SCA3 NSCs and identified 5 heterozygous SNPs in exons of the *ATXN3* gene (Fig. 1b). Two of these *ATXN3* SNPs (rs1048755 and rs910369, referred to as *ATXN3*_SNP2 and *ATXN3*_SNP5) were selected to design ddPCR assays. These SNPs either lead to missense mutation in N-terminal ubiquitin-interacting motif 1 (UIM1) of ataxin-3 [26] or are considered to cause alterations of RNA-binding protein (RBP)-binding sites in *ATXN3* mRNA [27].

#### Validation of the ATXN3 ddPCR assay specificity and accuracy

To examine specificity of allelic discrimination of *ATXN3* ddPCR assays, we performed *ATXN3*_SNP2 and *ATXN3*_SNP5 ddPCRs on predefined mixtures of plasmids containing either WT or MUT sequences with respective SNPs. We observed that the ratio of the signal originating from WT and MUT probes was extremely consistent with the ratio of premixed WT/MUT plasmids for both SNPs: SNP2 (Fig. 1c) and SNP5 (Additional File 1: Fig. S1A). To further validate the *ATXN3* ddPCR accuracy, we examined gDNA isolated from SCA3 fibroblasts, iPSCs, NSCs, and neurons. We used the *ATXN3*_SNP5 assay that utilizes the SNP variant located in the 3′UTR of the *ATXN3* gene, rendering the assay suitable for gDNA analysis. We found that in all analyzed gDNA samples, the WT/MUT allele ratio was remarkably close to the expected 50:50 (Fig. 1d).

#### Determination of the ATXN3 mRNA allele ratio in SCA3 cell lines

**Fig. 2:**
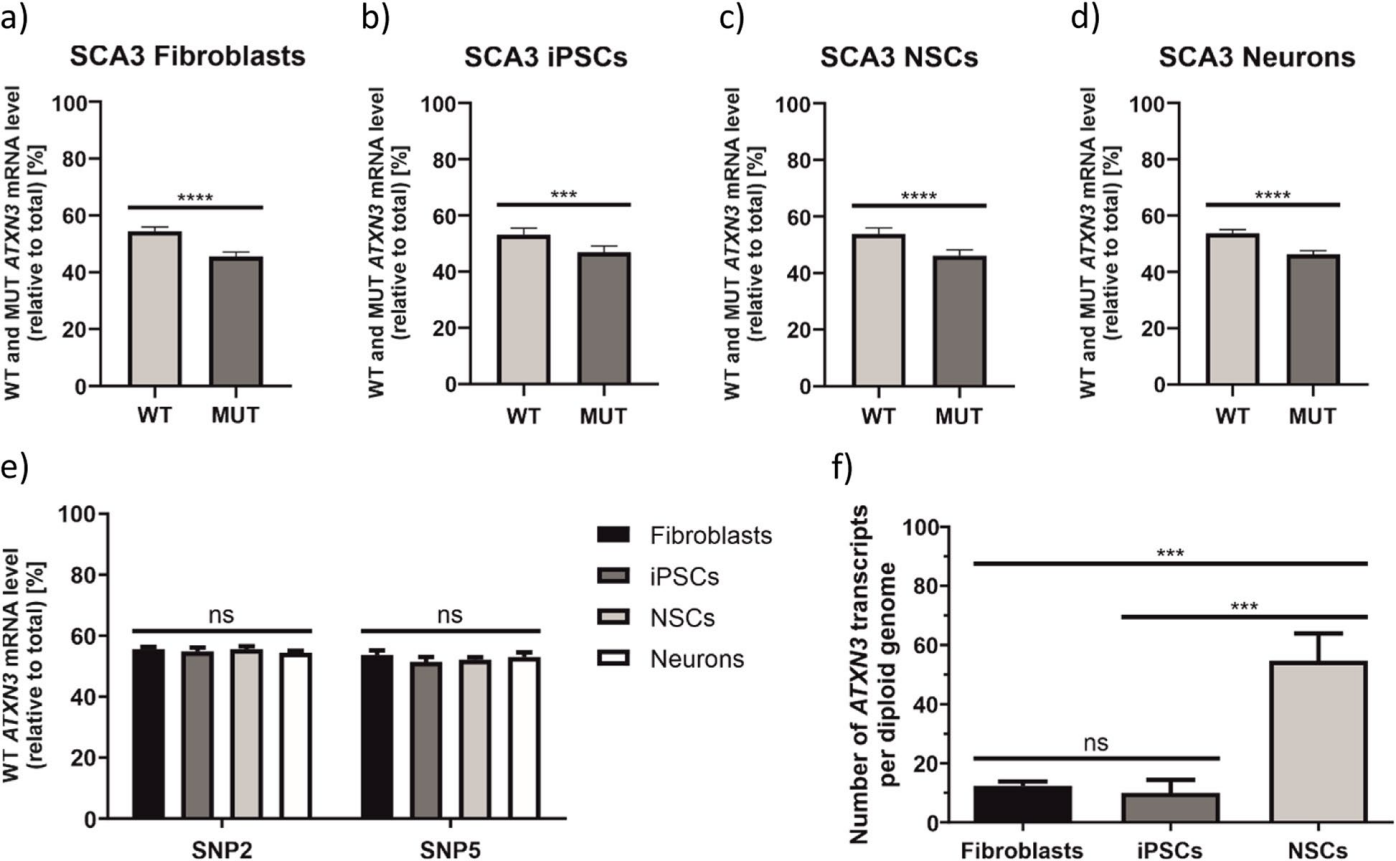
Determination of endogenous WT and MUT ATXN3 allele expression ratios in SCA3 cell lines. **a–d** Results from ddPCR are presented as a mean WT and MUT ATXN3 transcript allele abundance, calculated based on results from both ATXN3 assays (ATXN3_SNP2 and ATXN3_SNP5), in patient-derived fibroblasts (**a**), iPSCs (**b**), NSCs (**c**), and neurons (**d**). These data were analyzed using unpaired t test. **e** Corresponding results to **a–d** presented for ATXN3_SNP2 and ATXN3_SNP5 assays separately and analyzed with two-way ANOVA with Tukey’s multiple comparison test for identification of any cell type-specific changes. In this graph, only WT allele abundance is presented, the MUT allele abundance equals a remainder to the sum of 100%). **f** Estimation of the total number (WT + MUT) of endogenous ATXN3 transcripts per diploid genome using ATXN3_SNP5 assay. Data were analyzed using one-way ANOVA with Tukey’s multiple comparison test. For all experiments presented in this figure *n*=3. Two-tailed *p* value < 0.05 was considered significant and is depicted in the figure by: *p < 0.05; **p < 0.01; ***p < 0.001; ****p < 0.0001. All data are presented as means ± SD. Individual data values are available in Additional File 13

We examined allele-specific expression patterns of *ATXN3* in SCA3 cell lines: patient-derived fibroblasts, iPSCs reprogrammed from fibroblasts, as well as NSCs and neurons differentiated from iPSCs (Fig. 1a). To confirm cellular identity of generated neurons, we performed immunostaining and observed strong expression of *MAP 2* and *TUJ1* neuronal markers (Additional File 2: Fig. S2). When we quantified the *ATXN3* allelic expression ratios in SCA3 cell lines, we observed 54.7% WT/45.3% MUT ratio in fibroblasts, 53.2% WT/46.8% MUT ratio in iPSCs, 53.9% WT/46.1% MUT in NSCs, and 53.8% WT/46.2% MUT ratio in neurons (Fig. 2a–d, precise values for each *ATXN3* assay are given in Additional File 3: Fig. S3). We detected significantly higher expression of the WT allele, as compared to MUT allele, in almost all analyzed cell types. Two-way ANOVA showed that there are no statistically significant changes in the WT abundance between cell types (Fig. 2e); therefore, we conclude that the ratio of endogenous *ATXN3* alleles is not influenced by the cell type (in analyzed set of cell lines).

#### Estimation of the number of endogenous ATXN3 transcripts per diploid genome

To examine whether there are differences in the total number of *ATXN3* transcripts among analyzed cell lines, we split cells into two tubes and performed concurrent RNA and DNA isolations. We used the 3′UTR-located *ATXN3*_SNP5 assay to analyze both cDNA and gDNA. By dividing the total copy number of *ATXN3* transcripts by the total copy number of gDNA amplicons, we estimated a number of *ATXN3* transcripts per diploid genome. We documented approximately 12, 10, and 54 copies of *ATXN3* mRNA in fibroblasts, iPSCs, and NSCs, respectively (Fig. 2f). This result suggests the increase of *ATXN3* expression in the process of neural differentiation. Moreover, the number of *ATXN3* transcripts calculated for fibroblasts corresponds well with our previous results generated using single-molecule FISH, for this cell line (approximately 18 copies of *ATXN3* mRNA per cell) [28].

### Examination of endogenous HTT transcripts in cell lines

**Fig. 3:**
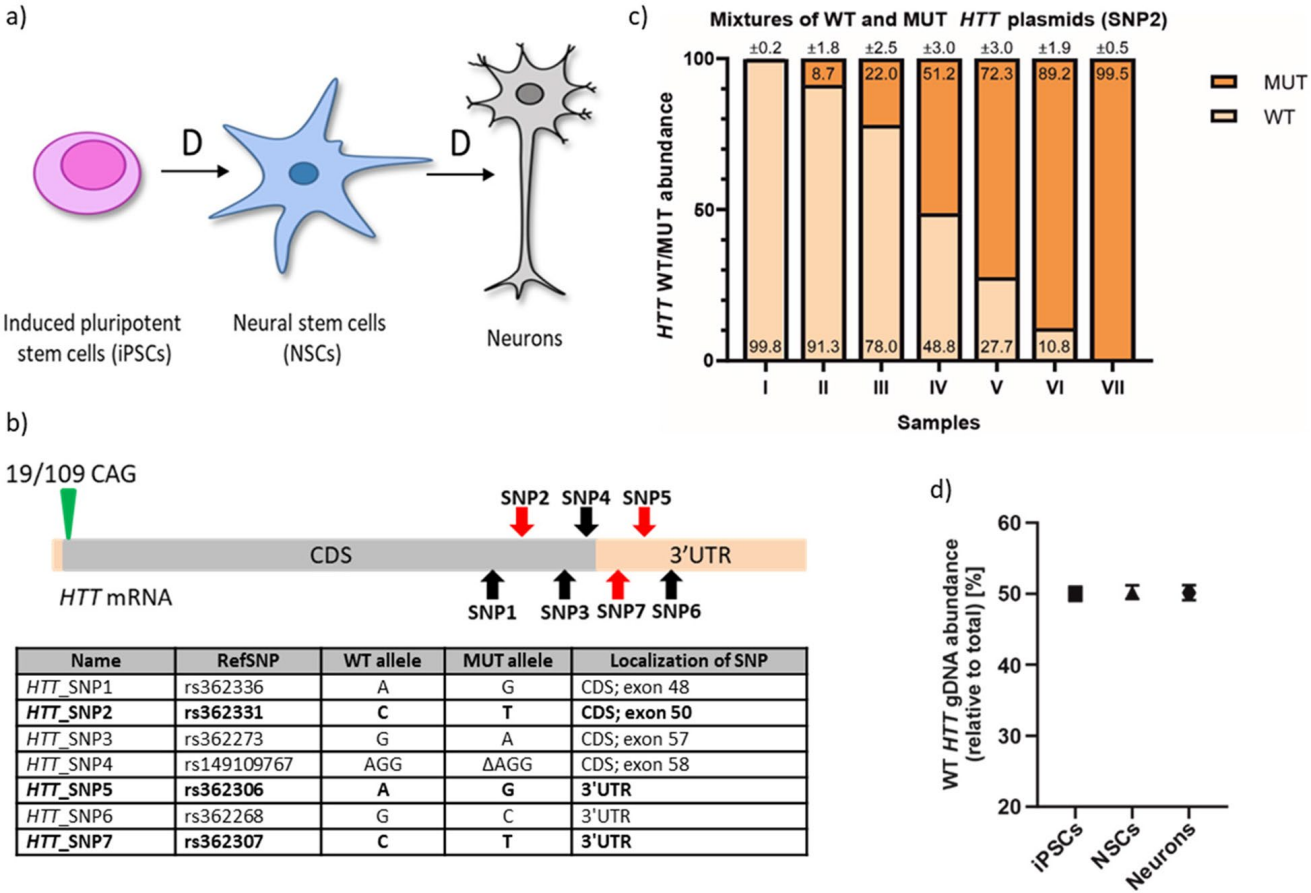
Assessing the specificity of HTT ddPCR assays. **a** HD cell lines used; D—differentiation. **b** Scheme of HTT transcript with marked CAG repeat tract (green triangle) and SNP variants identified in HD cell lines (red arrows—SNPs used in ddPCR assays; black arrows—other identified SNPs); light orange box—UTRs. Table presents all SNP variants identified in analyzed HD cell lines. Bolded SNPs were selected to be targets for ddPCR assays and they will be hereinafter referred to as HTT_SNP2, HTT_SNP5, and HTT_SNP7 in the text. CDS—coding sequence, ∆—deletion. RefSNP number according to the Single Nucleotide Polymorphism Database (dbSNP). **c** Results from ddPCR analysis of HTT_SNP2 assay specificity performed on seven samples with predefined ratios of WT/MUT plasmids (samples: I—100% WT and 0% MUT; II—90% WT and 10% MUT; III—75% WT and 25% MUT; IV—50% WT and 50% MUT; V—25% WT and 75% MUT; VI—10% WT and 90% MUT; VII—0% WT and 100% MUT). Precise values are indicated on WT/MUT bars ± poisson error. **d** Percentage ratio of HTT WT allele obtained with ddPCR HTT_SNP7 assay using gDNA from indicated HD cell lines. Three biological replicates were performed. These data are presented as means ± SD

We used a set of patient-derived cell lines with the HD genetic background: iPSCs, NSCs, and neurons (characterized by 19/109 CAG repeats in *HTT*) for ddPCR assay discriminating between expression of *HTT* WT and MUT allele (Fig. 3a).

#### Identification of SNP variants in HTT transcripts

To identify SNP variants located in *HTT* transcripts, we replicated our approach implemented for *ATXN3*. First, through the cloning and sequencing process, we identified 7 heterozygous SNPs in *HTT* exons in HD patient-derived NSCs (Fig. 3b). Three of them (rs362331, rs362306, and rs362307, named *HTT*_SNP2, *HTT*_SNP5 and *HTT*_SNP7, respectively) were selected for design of ddPCR assays (Fig. 3b). Those SNPs were described as associated with the most common *HTT* haplotype [29], correlated with the presence of mutation in *HTT* allele [30], and/or were targets in clinical testing of therapeutic approaches using ASOs.

#### Validation of specificity and utility of the HTT ddPCR assays

To assess allelic specificity of the HD ddPCR assay, we prepared plasmid mixtures of predefined WT/MUT ratios. We confirmed that ddPCR output accurately reflects tested plasmid ratios, using all three assays: *HTT*_SNP2 assay (Fig. 3c), as well as for *HTT*_SNP5 and *HTT*_SNP7 assays (Additional File 1: Fig. S1B and C). To determine the reliability of the HD ddPCR, we performed *HTT*_SNP7 assay (located in 3′UTR) on gDNA isolated from HD iPSCs, NSCs, and neurons. Amplification of *HTT* gDNA alleles from HD cells showed a canonical 50/50 WT/MUT ratio (Fig. 3d).

#### HTT WT/MUT ratio determination in HD cell lines

**Fig. 4:**
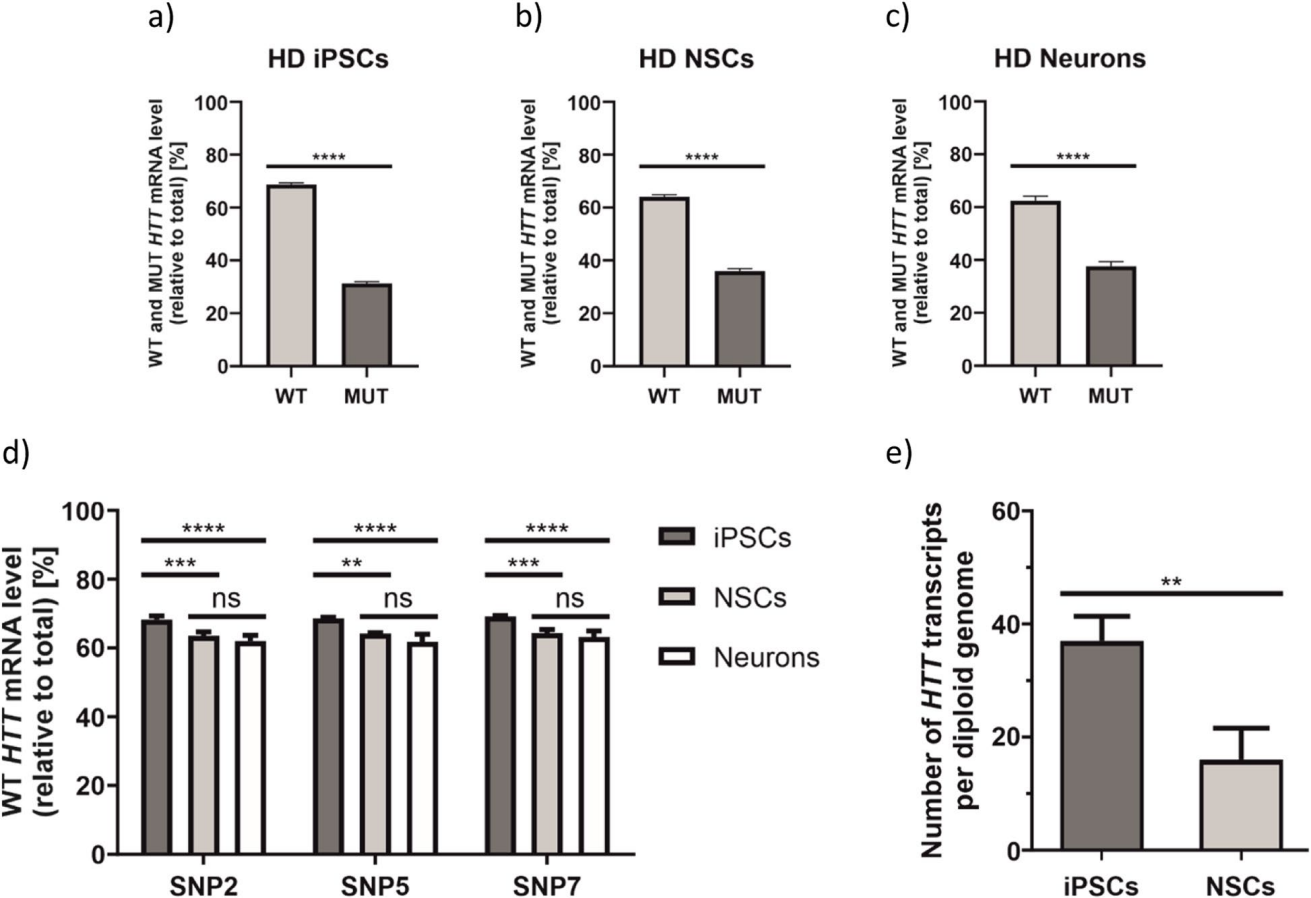
Determination of endogenous WT and MUT HTT allele expression ratios in HD cell lines. **a*–c*** Results from ddPCR are presented as a mean WT and MUT HTT transcript allele abundance, calculated based on results from all three HTT assays (HTT_SNP2, HTT_SNP5 and HTT_SNP7), in iPSCs (***a***), NSCs (***b***), and neurons (***c***). These data were analyzed using unpaired *t* test. ***d*** Corresponding results to ***a–c*** presented for HTT_SNP2, HTT_SNP5, and HTT_SNP7 assays separately and analyzed with two-way ANOVA with Tukey’s multiple comparison test for identification of any cell type-specific changes. In this graph, only WT allele abundance is presented, the MUT allele abundance equals a remainder to the sum of 100%). ***e*** Estimation of the total number (WT + MUT) of endogenous HTT transcripts per diploid genome using HTT_SNP5 assay. Data were analyzed using unpaired *t* test. For all experiments presented in this figure *n*=3. Two-tailed *p* value < 0.05 was considered significant and is depicted in the figure by: *p < 0.05; **p < 0.01; ***p < 0.001; ****p < 0.0001. All data are presented as means ± SD. Individual data values are available in Additional File 13

To verify allelic expression of the *HTT* gene, we examined HD iPSCs, NSCs, and neurons using *HTT*_SNP2, *HTT*_SNP5, and *HTT*_SNP7 ddPCR assays. Cellular identity of generated neurons was confirmed by immunostaining for expression of *DARPP32*, *TUJ1*, *MAP 2,* and *GAD67* neuronal markers (Additional File 2: Fig. S2). Allelic expression ratio in iPSCs averaged 68.7% WT/31.3% MUT (Fig. 4a; precise values for each *HTT* assay are given in Additional File 4: Fig. S4). In NSCs, we observed a slightly lower WT allele contribution, resulting in 64% WT/36% MUT ratio (Fig. 4b). In HD neurons, WT allele fraction dropped even further to 62.4% WT/37.6% MUT (Fig. 4c). The differences in allelic ratios between various HD cell lines were statistically significant (Fig. 4d). Comparing results obtained from HD and SCA3 cell lines, we documented greater differences between WT/MUT allele ratio in HD cells, than in SCA3 cells. We also noticed that the abundance of the MUT *HTT* allele increases with neural differentiation process (Fig. 4d).

To directly compare ddPCR results with information obtained from RNA-Seq, we extracted *HTT*-relevant reads from data obtained for HD iPSCs and NSC transcriptomes. We looked at *HTT* reads that span the sequences of three SNP variants selected for ddPCR design. The allelic expression ratio in iPSCs averaged 74.7% WT/25.3% MUT (Additional File 5: Fig. S5A), while in NSCs we obtained the ratio of 73.8% WT/26.2% MUT (Additional File 5: Fig. S5B), and these results were roughly consistent with ddPCR results. However, when each SNP region (SNP2, SNP5 or SNP7) was analyzed separately, we observed substantial differences in WT/MUT allele ratio, in both cell lines (Additional File 5: Fig. S5C-E), which generated higher error bars during statistical analysis. This implies that ddPCR is a more accurate and precise method for such approach.

#### Estimation of the number of endogenous HTT transcripts per diploid genome

To estimate an absolute number of *HTT* transcripts per diploid genome, we analyzed cDNA and gDNA from iPSCs and NSCs using *HTT*_SNP5 and *HTT*_SNP7 assays. As in SCA3 experiments, we divided the total number of *HTT* transcripts by the number of gDNA amplicons and, as a result, ~37 *HTT* transcripts in iPSC and ~16 *HTT* transcripts in an NSC were calculated for both *HTT*_SNP5 (Fig. 4e) and *HTT*_SNP7 assays (Additional File 6: Fig. S6). The difference between the number of *HTT* mRNAs in iPSCs and NSCs was statistically significant. Furthermore, the number of *HTT* transcripts calculated for NSCs corresponds well with our previous estimation obtained for this cell line using smFISH (~17 *HTT* mRNA molecules per cell) [28].

#### Exploration of WT/MUT HTT allelic expression after gene editing in iPSCs

**Fig. 5:**
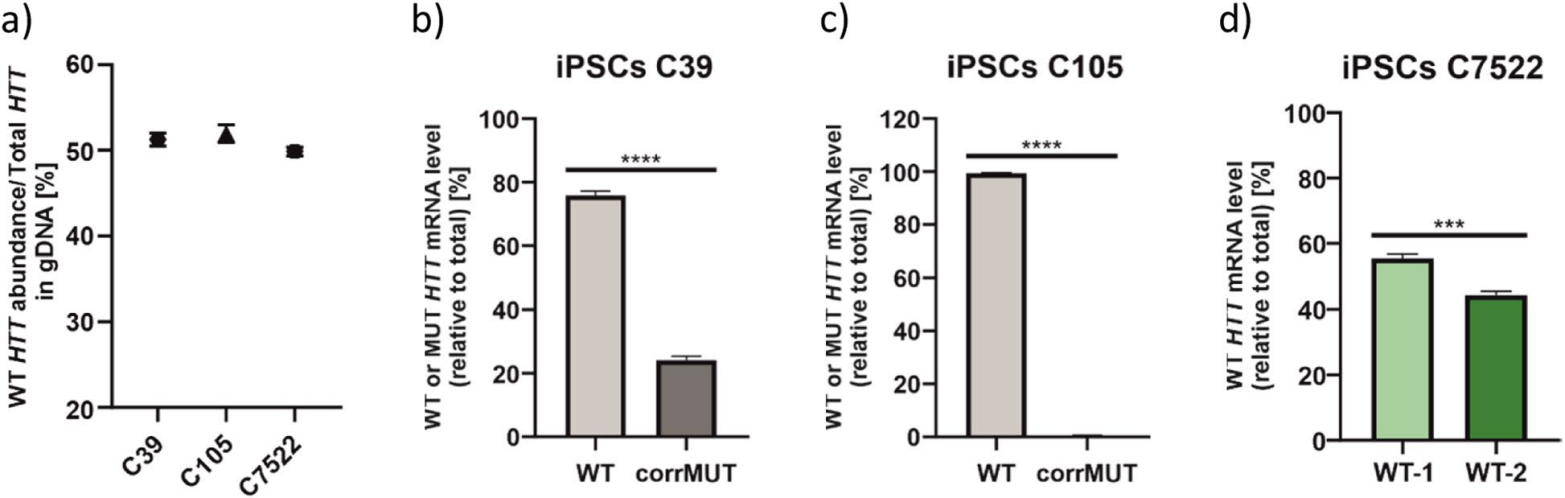
Determination of endogenous HTT allele ratios in genetically corrected HD iPSCs and non-HD iPSCs. **a** Percentage ratio of HTT WT allele obtained with ddPCR HTT_SNP7 assay (or HTT_SNP5 regarding C7522 cells) using gDNA from indicated iPSC lines. ***b,c*** Results from ddPCR are presented as a mean WT or corrMUT HTT transcript allele abundance, calculated based on results from all three HTT assays (HTT_SNP2, HTT_SNP5, and HTT_SNP7), in genetically corrected C39 (***b***) and C105 (***c***). ***d*** Ratio of WT alleles of HTT transcript (WT-1 and WT-2) in control C7522 cell line. Data were analyzed using unpaired *t* test. For all experiments presented in this figure *n*=3. Two-tailed *p* value < 0.05 was considered significant and is depicted in the figure by: *p < 0.05; **p < 0.01; ***p < 0.001; ****p < 0.0001. All data are presented as means ± SD. Individual data values are available in Additional File 13

We previously used a CRISPR-Cas9 method to correct the CAG expansion in HD iPSCs, and we generated isogenic control lines C39 and C105 [31]. Here, we inspected these lines to precisely determine expression of WT and corrected MUT (corrMUT) alleles. Additionally, we used a non-HD-related iPSC line derived from a healthy individual (C7522) that is also heterozygous for SNP5. When we examined gDNA of all three cell lines, we found nearly perfect 50/50 allelic ratio, which indicates that in the case of C39 and C105 lines CRISPR-Cas9 editing did not affect analyzed genomic sequences of the *HTT* gene (Fig. 5a). Our analyses of *HTT* allelic expression performed in C39 and C105 lines showed significantly altered expression in comparison with the initial iPSC line. While the initial iPSC line averaged 68.7% WT/31.3% MUT (Fig. 4a), C39 showed the average ratio of 75.9 WT/24.1% corrMUT (Fig. 5b; precise values for each *HTT* assay are given in Additional File 7: Fig. S7) and C105 revealed a ratio of 99.5 WT/0.5% corrMUT (Fig. 5c). All three SNP assay measurements for C39 and C105 were consistent (Additional File 7: Fig. S7), confirming almost total silencing of expression of the corrMUT allele in C105 line. Results obtained for C105 line suggest that there are off-target effects of gene editing that affected the expression of corrected allele, whereas the change in ratio of WT/corrMUT alleles in C39 line may result from CAG repeat tract shortening or, also, from some off-target effects. In control C7522 iPSC line, *HTT*_SNP5 assay showed 55.6% [WT-1]/44.4% [WT-2] ratio (Fig. 5d), suggesting more equal ratio in case of two WT alleles.

#### Silencing of endogenous HTT expression in HD NSCs

**Fig. 6:**
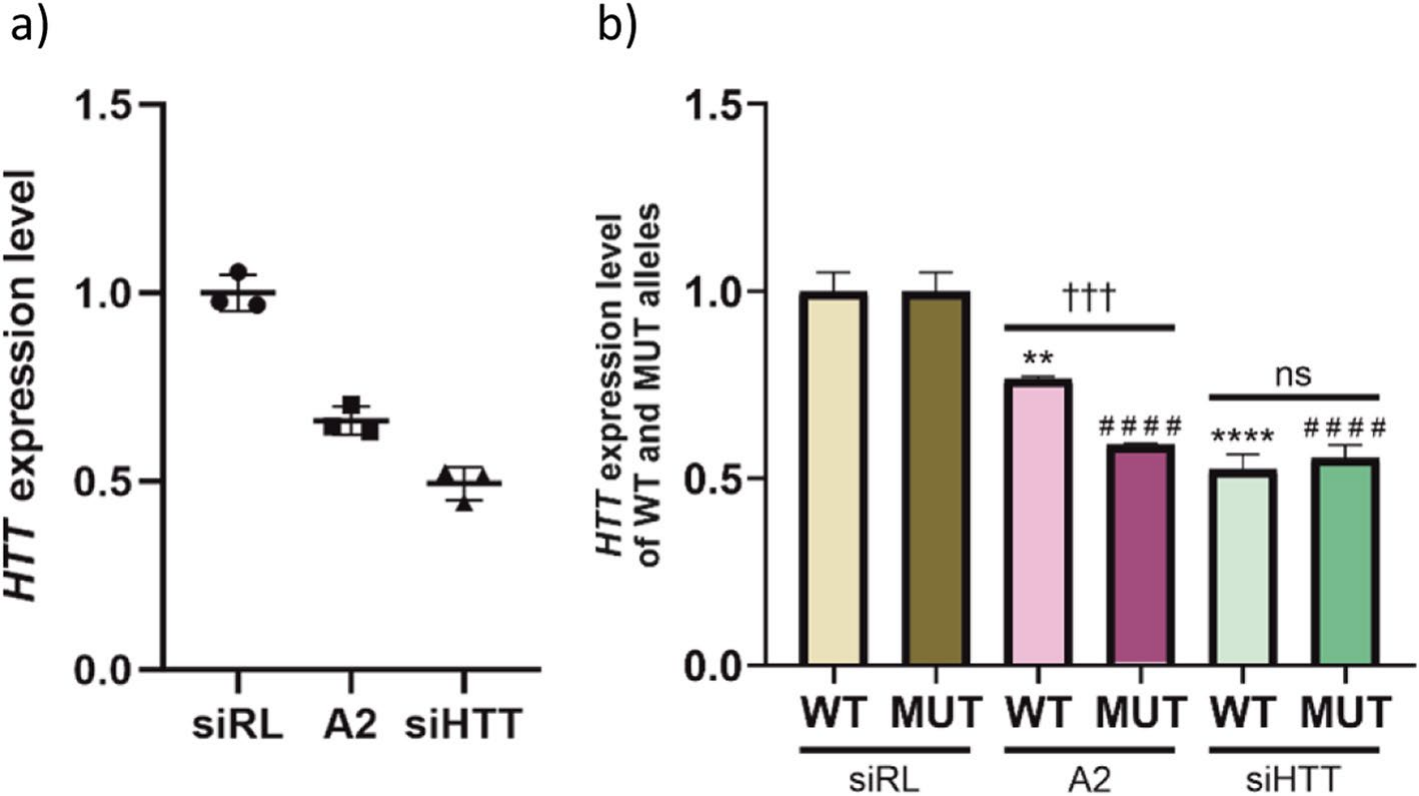
Silencing of endogenous HTT expression in HD NSCs. **a** Endogenous HTT expression level after HD NSCs transfection with indicated oligonucleotides (siRL, A2, siHTT) assessed using ddPCR. ***b*** HTT WT and MUT allele abundance after transfection with indicated oligonucleotides. Results in ***a*** and ***b*** were normalized to HTT expression level in cells transfected with control siRNA targeting Renilla luciferase (siRL). Mean values from results obtained using HTT_SNP2, HTT_SNP5, and HTT_SNP7 are presented. Two-way ANOVA with Tukey’s multiple comparison test was used to analyze changes in HTT expression level [WT (*) or MUT (#) allele], while unpaired *t* test was used to determine significance between WT and MUT HTT allele downregulation for each oligonucleotide (†). For all experiments presented in this figure *n*=3. Two-tailed *p* value < 0.05 was considered significant and is depicted in the figure by: *p < 0.05; **p < 0.01; ***p < 0.001; ****p < 0.0001. All data are presented as means ± SD. Individual data values are available in Additional File 13

To verify the *HTT* ddPCR assay in assessing the efficacy of allele-selective gene silencing, we transfected HD NSCs with selected oligonucleotides, such as allele-selective miRNA-like oligonucleotide (A2) targeting expanded CAG tracts [32], non-allele-selective siRNA (siHTT) targeting *HTT* mRNA [33], and non-target control siRNA (siRL) targeting *Renilla* luciferase [28]. A2 oligo and siHTT reduced overall *HTT* expression by around 35% and around 50%, respectively, as measured by ddPCR (Fig. 6a). When we looked at *HTT* allelic expression, we observed equal downregulation of WT and MUT alleles in cells transfected with siHTT. However, silencing of the MUT allele expression was more prominent, as compared to WT allele, in cells transfected with A2 (Fig. 6b). This confirms that siHTT and A2 functions, in gene silencing mechanisms, in a non-allele-selective and an allele-selective manner, respectively [28], and highlights the utility of the HD ddPCR assay as a precise tool for determining the efficacy of transcript-reducing therapeutic strategies.

### Examination of HTT transgenes in HD mice brain tissue

#### Determination of the HTT transgene copy number

**Fig. 7:**
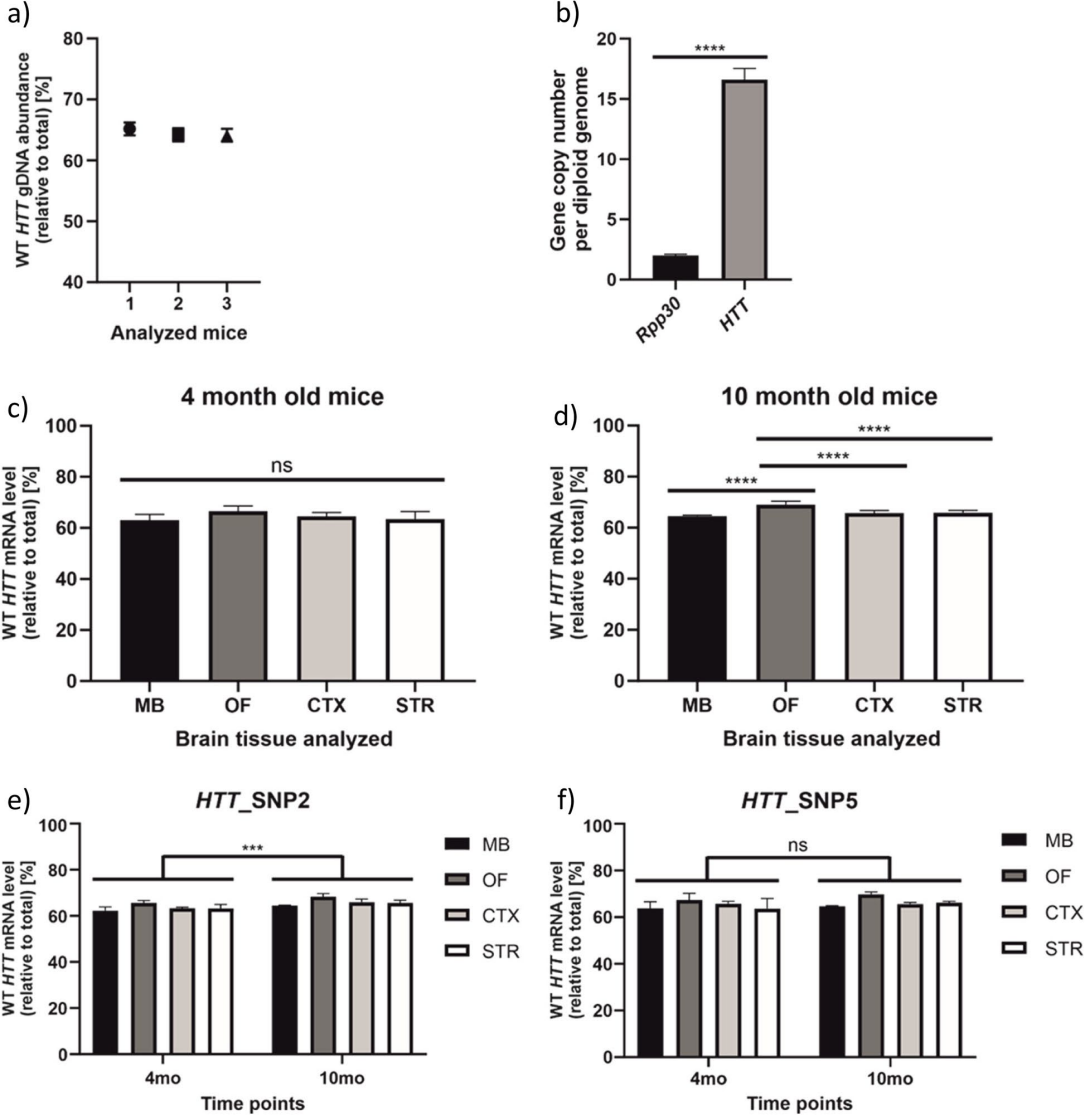
Analyses on brain tissues of Hu128/21 mice aged 4 and 10 months. **a** Abundance of *HTT* transgene WT allele determined using *HTT*_SNP5 ddPCR assay on gDNA obtained from three Hu128/21 mice. **b** Copy number of *HTT* transgene in Hu128/21 mice, calculated using *HTT*_SNP7 assay, compared to the reference gene *Rpp30.* **c,d** *HTT* WT transcript abundance in midbrain (MB), olfactory bulb (OF), cortex (CTX), and striatum (STR) of 4-month-old (**c**) and 10-month-old (**d**) Hu128/21 mice. **e,f** Comparison of *HTT* WT transcript abundance in analyzed brain tissues between 4- and 10-month-old mice according to *HTT*_SNP2 assay (**e**), and according to *HTT*_SNP5 assay (**f**). Data were analyzed using unpaired t test (**b**), ordinary one-way ANOVA (**c, d**), or two-way ANOVA (**e, f**) with Sidak multiple comparison test. In **e**, we present results obtained by two-way ANOVA analysis, where all 4 tissues were grouped together and analyzed by the significance of the time point factor. For all experiments presented in this figure *n*=3. Two-tailed p value < 0.05 was considered significant and is depicted in the figure by: *p < 0.05; **p < 0.01; ***p < 0.001; ****p < 0.0001. All data are presented as means ± SD. Individual data values are available in Additional File 13

To demonstrate the robustness of our HD ddPCR assay, we examined brain tissue from Hu128/21 mice, the HD mouse model created by crossing BAC21 and YAC128 mouse lines [34]. Hu128/21 mice carry *HTT* transgenes with heterozygous SNP variants for rs362331 (*HTT*_SNP2) and rs362306 (*HTT*_SNP5), allowing us to determine copy number and expression of mutant and wild type alleles. In the first step, we analyzed gDNA isolated from Hu128/21 mice using *HTT*_SNP5 assay. As BAC and YAC *HTT* transgenes are present in various copy numbers, the assay in mice did not show 50/50 ratio. Instead, we documented a mean 64.5% WT/35.5% MUT ratio (Fig. 7a). Next, to determine precise transgene copy number in Hu128/21 mice, we performed a copy number variation (CNV) assay. We performed ddPCR for *Rpp30* as a reference and used *HTT*_SNP7 assay targeting *HTT* transgene. Since the rs362307 (*HTT*_SNP7) SNP variant is homozygous in alleles of the transgene in Hu128/21 mouse model, this assay recognizes total *HTT*, without WT/MUT discrimination. Our results showed that *HTT* transgene was around 8.5 times more abundant than a reference gene (represented by two copies); thus, each mouse had around 17 copies of *HTT* integrated into its genome (Fig. 7b). Taking WT/MUT ratio into consideration, Hu128/21 mice had around 11 copies of BAC21 transgene and around 6 copies of YAC128 transgene.

#### HTT WT/MUT ratio determination in selected brain tissues of two age groups of Hu 128/21 mice

Next, we analyzed WT/MUT allelic expression in striatum (STR), cortex (CTX), midbrain (MB), and olfactory bulb (OF) of presymptomatic and symptomatic Hu128/21 mice. In presymptomatic 4-month-old mice, we detected 63.4% WT/36.6% MUT ratio in STR, 64.5% WT/35.5% MUT ratio in CTX, 63.0% WT/37.0% MUT ratio in MB, and 66.5% WT/33.5% MUT ratio in OF (Fig. 7c; precise values for each *HTT* assay are given in Additional File 8: Fig. S8). When we quantified allelic expression in symptomatic 10-month-old mice, we obtained 65.9% WT/34.1% MUT ratio in STR, 65.8% WT/34.2% MUT ratio in CTX, 64.6% WT/35.4% MUT ratio in MB, and 69.1% WT/30.9% MUT ratio in OF (Fig. 7d). The allelic expression ratios matched the allelic distribution found using genomic DNA, suggesting that differences in transgene expression result directly from their copy numbers. Moreover, performed quantifications of *HTT* transcripts are consistent with reported WT/MUT huntingtin protein levels in the original paper describing Hu128/21 mice [34]. Using two age groups of Hu128/21 mice, we aimed to investigate any age-related changes in *HTT* transgene expression ratios in selected tissues (Fig. 7e, f). Two-way ANOVA performed on the data obtained for *HTT*_SNP2 assay and all four tissues considered/analyzed together showed that WT *HTT* mRNA abundance in 10-month-old mice was slightly higher, when compared to 4-month-old-mice (Fig. 7e), suggesting consistent increase of WT/MUT expression ratio in time in analyzed tissues. However, when all tissues were analyzed individually, post hoc test did not show a significance.

There was no statistically significant time point-dependent changes of *HTT* allele expression ratio in test performed for particular tissue. However, according to the two-way ANOVA performed on the data obtained for *HTT*_SNP2 assay and all four tissues considered together, WT *HTT* mRNA abundance in 10-month-old mice was slightly higher, as compared to 4-month-old mice (Fig. 7e), suggesting consistent increase of WT/MUT expression ratio in time in analyzed tissues.

## Discussion

Three decades of extensive research have uncovered polyQ toxicity as a main driver of CAG repeat expansion diseases. However, a growing body of evidence suggests that CAG expanded transcripts also contribute to the pathogenesis and progression of these disorders [35, 36]. Investigation of RNA toxicity in dominantly inherited diseases is challenging since mRNA molecules transcribed from two nearly identical alleles of the causative genes cannot be easily distinguished. Since the polyQ disease genes are typically expressed at low levels, an ideal allele differentiating analytical method should demonstrate high accuracy, precision, sensitivity, and specificity. Because only a limited number of allele discrimination studies have been conducted in the context of transcripts related to polyQ diseases [37, 38], here, we developed an SNP-based ddPCR approach for allele-selective analyses of endogenous *HTT* and *ATXN3* transcripts. We performed a series of control amplifications using newly designed sets of primers and probes to show that our method is reliable and quantitative for analyzing low abundant transcripts with relatively complex GC-rich sequences. Analyses of the *ATXN3* and *HTT* transcripts in cells and tissues relevant to pathology of examined diseases, using independent SNP assays, demonstrated excellent reproducibility of transcript detection and discrimination.

The occurrence of differences in expression between two alleles of one gene is a known phenomenon [39], and several factors responsible for affecting mRNA allelic ratios were already identified using high-throughput analyses [40]. Interestingly, the presence of specific SNP variants itself can affect the expression level of alleles, e.g., through regulation of promoter activity, as shown for *HTT* [41], or changes in miRNA-binding sites in 3′UTR, which was recently shown for *ATXN3* expression [27]. Our allelic expression analysis in several HD and SCA3 cellular and mouse models revealed significant differences between WT and MUT alleles. These differences in selected patient-derived cell lines were generally lower in SCA3 cells (~54% WT/46% MUT) than in HD cells (62–69% WT/31–38% MUT) (Figs. 2e and 4d), but more cell lines should be used to draw general conclusions about *ATXN3* and *HTT* expression in patients’ cells. Such broader analyses were performed on postmortem HD brain samples previously, using standard quantitative RT-PCR, rather than more precise ddPCR [37]. In that study, in the majority of tested samples, MUT *HTT* RNA was more abundant than the WT; however, a precise WT/MUT allele ratio seems to be an individual case. Another interesting issue to examine in the future is alternative transcript variants of both genes, *ATXN3* and *HTT*. In this research, we wanted to focus on the main variants of each transcript, but analyses on, e.g., *HTT*intron1 variant [14, 42] or *ATXN3* transcripts which does not contain exon 11 (which lead to the translation of ataxin3-a long and ataxin3-a short proteins) [26], might be interesting in terms of revealing new facts regarding the pathogenesis of both diseases.

For both cell lines, SCA3 and HD, we showed that the total number of the *ATXN3* and *HTT* transcripts, respectively, changes during neuronal differentiation in cell culture conditions (Figs. 2f and 4e). Estimations of examined transcripts per diploid genome showed diverse trends, which we confirmed using various calculation approaches. Moreover, these results corresponded well with smFISH-based detection of *ATXN3* and *HTT* in SCA3 fibroblasts and HD NSCs, respectively [28]. Interesting observations were made by Didiot et al., concerning normal *HTT* mRNA abundance and localization [43]. Not only they showed that the number of *HTT* mRNAs decreased with reprogramming of human fibroblasts into neuron-like cells, but also the localization of *HTT* mRNA changed—in neuron-like cells, *HTT* transcript was detected more often in the nuclear fraction, than in the cytoplasmic one. This is an interesting issue for further examination using both SCA3 and HD cells, together with discrimination of normal and mutant mRNA alleles. Additionally for *HTT*, we showed that abundance of MUT allele vs. WT allele increases with neuronal differentiation (Fig. 4d), what was also shown previously at a protein level in mouse cell lines obtained from HD YAC128 model [44, 45]. Again, these observations require confirmation in a larger set of cell lines, e.g., for investigation of processes that might be related to the phenomenon of neuronal vulnerability to mutations causing polyQ diseases [46]. However, these results support the idea that presented approach can be applied in studies aimed at the investigation of RNA biology of mutant transcripts leading to a better understanding of the pathogenesis of polyQ disorders [4, 5, 47–49]. Additionally, our approach could be also used for analyses of non-neuronal cells, as these cells were reported to be implicated in the pathogenesis of both SCA3 and HD [4, 50, 51]. Moreover, a joint characteristic for polyQ diseases is a regional vulnerability of specific brain regions to neurodegeneration. Hence, our assays could be used in determining whether specific cells (e.g., cortical neurons, Purkinje cells) show varied WT/MUT ratios or absolute numbers of transcripts per cell.

We applied our ddPCR-based approach in the evaluation of genetic engineering methods. The CRISPR-Cas9 technology provides a powerful tool for cell line generation, but off-target effects may affect gene expression and have an impact on the obtained results [52]. During the examination of potential off-target effects, an analysis performed on gDNA may provide only limited information about how the introduced modification affects the expression of the gene-of-interest. On the other hand, qPCR on cDNA is not as precise as ddPCR—since the amplification is exponential (meaning a 2-fold difference in number of molecules per cycle), qPCR is prone to even minor pipetting errors or incorrect choice of a reference gene [53]. Using previously generated isogenic control iPSC lines [31], we showed that quantitation using ddPCR can overcome this potential limitation. Additionally, based on RNA-Seq data from HD iPSCs and NSCs, we were able to estimate the ratio of WT/MUT alleles, but the accuracy and precision of ddPCR-based quantitation was much higher.

The analyses performed with the use of Hu128/21 mice tissues confirmed that the approach can be very useful for the investigation of polyQ disease-related transcripts in animal models. Unlike cells, transgenic Hu128/21 mice carried unequal distribution of WT and MUT gene copies already in gDNA. Interestingly, we showed differences in *HTT* mRNA levels originating from WT vs. MUT copies between analyzed tissues in the 10-month-old mice. Despite the fact that mostly striatal and cortical neurons are affected in HD [5], we have not observed significant differences in *HTT* mRNA WT/MUT ratios in those tissues, as compared to other two analyzed. Statistical analysis also showed that there was a slightly higher WT mRNA allele abundance in 10-month-old mice in comparison to 4-month-old animals. This observation is consisted with results from human HD brain tissues, where lower abundance of WT mRNA allele (relative to MUT allele) was reported in samples from patients at early disease stages, as compared to samples from patients at late disease stages [37]. However, more testing is required to verify whether those observations are general or model-specific. So far, time course of mutant and total HTT protein levels was investigated in detail in BACHD rats, showing that the ratio between mutant and total huntingtin can significantly change in time [54].

There is still no available treatment for polyQ diseases. The experimental approaches which are considered as the most promising (as the most efficient and safe) for HD and SCA3 are based on allele-selective targeting of mutant alleles [55–58]. Preferential targeting of mutant transcripts requires precise evaluation of efficiency and selectivity of the used strategy, e.g., through quantitative analysis of WT and MUT protein levels [59–61]. However, protein assays usually require more tissue or CSF, which is often limited. The solution can be a highly sensitive transcript assay, like the one described in this study. Since measurements of the mutant gene expression level are also considered as important biomarker in polyQ diseases [37, 62, 63], our approach can be considered as a powerful tool in clinical research.

## Conclusions

We adapted a SNP-based ddPCR method for the evaluation of WT/MUT allele expression ratio in SCA3 and HD cells, as well as in the HD mouse model. Our results clearly show that the allele-specific ddPCR methods presented in this work is suitable for the detection of slight variations in the expression levels of tested alleles in both cell cultures and mice tissues implicated in polyQ diseases. We examined various applications where our approach can be used and, most importantly, we showed its usefulness for accurate and precise analysis of allelic expression during cell differentiation, and after gene editing, as well as in assessing the efficacy of polyQ therapies.

## Methods

### Cell lines and cell culture

SCA3 fibroblasts (GM06153, 16/67 CAG repeats in *ATXN3*) were obtained from the NIGMS Repository (Coriell Institute for Medical Research). They were cultivated in Minimum Essential Medium (Gibco), containing 10% fetal bovine serum (Biowest), a penicillin-streptomycin solution (Sigma-Aldrich), and 2mM L-glutamine (Sigma-Aldrich). SCA3 iPSCs were generated (from GM06153 fibroblasts) previously [64]. HD iPSCs (ND42222, 19/109 CAG repeats in *HTT*) were obtained from the NIGMS Repository. Additional cell lines were used: iPSC clonal lines (C39 and C105) previously generated via CRISPR-Cas9-based modification of the ND2222 line [31], and a non-HD-related iPSC line (C7522) generated from a healthy individual-derived fibroblast line (GM07522) obtained from the NIGMS Repository. iPSCs were cultured in StemFlex (Gibco) medium on Geltrex (Gibco). NSCs were grown in STEMdiff Neural Progenitors Medium (NPM) (STEMCELL Technologies).

### Differentiation of HD and SCA3 iPSCs to NSCs

iPSCs were differentiated to NSCs using the STEMdiff SMADi Neural Induction Kit (STEMCELL Technologies) and a monolayer protocol, following the manufacturer’s instructions. Briefly, iPSCs were grown on a 6-well plate until 70–80% confluence was reached, and then dissociated to single cells by incubation with Accutase (STEMCELL Technologies) for 4 min. Cells were counted using TC20 Automated Cell Counter (Bio-Rad) and resuspended at 1 × 10^6^ cells/ml density for seeding in STEMdiff Neural Induction Medium with SMADi and 10 nM Y-27632 (all from STEMCELL Technologies). After third passage, they were grown in STEMdiff NPM.

### Differentiation of SCA3 NSCs to neurons

SCA3 NSCs were differentiated to neurons according to protocol published by Hansen et al. [65] with slight modifications. Briefly, NSC were grown until full confluence and then dissociated to single cells by incubation with Accutase. Collected cells were passaged in 1:10 ratio onto Geltrex-coated 6-well and 12-well (for ICC) plates with NPM and 10 nM Y-27632. Next-day NPM was exchanged for neural maturation medium N2B27 with 20 ng/mL recombinant human BDNF and 10ng/mL recombinant human GDNF (both STEMCELL Technologies) and 300 ng/μL cAMP (Sigma-Aldrich). Medium was changed every 2 days for 3 weeks.

### Differentiation of HD NSCs to MSNs

HD NSC neural differentiation was performed according to protocol published by M. Fjodorova and M. Li [66] with slight modifications. Briefly, NSCs were grown in STEMdiff NPM until full confluence and then dissociated to single cells by incubation with Accutase. Collected cells were passaged in 1:5 ratio onto a Geltrex-coated 6-well plate with NPM and 10 nM Y-27632. Next-day NPM was exchanged for LGE pattering medium with 25 ng/mL recombinant human Activin A (STEMCELL Technologies). Medium was changed every day until day 10 when LGE progenitors were passaged for terminal differentiation into medium spiny neurons (MSNs): cells were passaged with Accutase in ratio 1:5 onto Geltrex precoated 6-well and 12-well plates (for ICC) in LGE pattering medium and 10 nM Y-27632. Next-day LGE pattering medium was exchanged for a terminal differentiation medium N2B27 with 10 ng/mL recombinant human BDNF and 10 ng/mL recombinant human GDNF (both STEMCELL Technologies). Medium was changed every 2 days for 3 weeks.

### Immunocytochemistry (ICC)

NSC and neuronal cells were fixed firstly for 5 min by adding 2% PFA directly to a cell culture medium and then gently washed in PBS followed by fixation in 4% PFA in PBS for 10 min. Cells were permeabilized with 0.5% Tween, blocked in 1% bovine serum albumin, and incubated with primary antibodies and fluorescent-dye conjugated secondary antibodies (listed in Additional File 9: Table S1). DAPI was used for DNA staining. Images were captured with a Leica DMI6000 microscope (Additional File 10: Fig. S2).

### HD mice

Hu128/21 HD mice model was described previously [34]. Briefly, the Hu128/21 mouse model was created by crossing Hdh−/− BAC21 mice (Hu21) and Hdh−/− YAC128 mice (Hu128), and then genotyped. Mice were housed in the animal facility of the Center for Advanced Technology (CAT) in Poznań and kept under standard conditions with a 12-h light/dark cycle with water and food ad libitum. All procedures and handling of the animals were performed to minimize stress to the animals and according to a protocol approved and monitored by the Local Ethical Commission for Animal Experiments in Poznan (Poland). Mice were sacrificed and brain tissues harvested and snap frozen in liquid nitrogen and stored at −80°C. Number and age of mice used in all experiments are reported in the accompanying figure legends.

### SNP variant identification

For SNP variant identification, the TOPO XL-2 PCR Cloning Kit (Invitrogen) was used. Briefly, RNA isolated from SCA3 and HD cell lines (iPSCs) was reversely transcribed using High-Capacity cDNA Reverse Transcription kit (Applied Biosystems) with random hexamer primers (for *ATXN3*) or SuperScript IV Reverse Transcriptase (Thermo Fisher) with a gene-specific primer (HTT_RT) (for *HTT*). Then, cDNA served as a template in PCR reactions using Platinum SuperFi polymerase included in a cloning kit. In case of *HTT*, PCRs were performed using PrimeSTAR GXL DNA Polymerase (Takara Bio). Primers in those PCR reactions were designed in a way to amplify whole CDSs, with fragments of both 5′ and 3′UTRs (ATXN3_Fwd and ATXN3_Rev; HTT_Fwd and HTT_Rev, sequences given in Additional File 10: Table S2). PCR products were then cloned into pCR-XL-2-TOPO vector. Generated vectors were transformed into bacterial cells provided with the kit. Transformants were analyzed through colony PCR and restriction enzyme digestion to select clones with either WT or MUT versions of analyzed genes. Positive clones were then sequenced, and SNPs allowing for discrimination between WT and MUT alleles, after additional confirmation on genomic DNA (gDNA), were identified. All identified SNPs are listed in Figs. 1b and 3b.

### RNA isolation and reverse transcription

For RNA isolation, the Total RNA Zol-Out D kit (A&A Biotechnology) was used, according to the manufacturer’s protocol. In case of cells, cell pellets were suspended in 800 μL of TRI Reagent (Thermo Fisher), while mouse tissues were homogenized in 300 μL of TRI Reagent. The concentration of isolated total RNA was calculated by measurement at 260 nm using DeNovix spectrophotometer. One microgram of RNA was then used for reverse transcription using the High-Capacity cDNA Reverse Transcription kit (Applied Biosystems) with random hexamer primers.

### Genomic DNA isolation

gDNA was isolated using the Genomic DNA Isolation kit (Norgen Biotek Corp.) according to the manufacturer’s protocols designed for cells growing in a monolayer and animal tissues. The concentration of isolated gDNA was calculated by measurement at 260 nm using the DeNovix spectrophotometer.

### Droplet digital PCR

Among various ddPCR applications, Rare Mutation and Sequence Detection were chosen. To specifically target sequences differing by only one nucleotide, two probes labelled with different fluorophores were designed together with a pair of primers. Because of the high dilution of the sample, probes bind to templates carrying specific SNP variants with a high specificity. After the readout based on the fluorescence, data were analyzed using Poisson statistics to determine the concentration of template carrying each SNP variant (and its flanking sequence) in the original sample. ddPCRs were prepared using DG8 cartridges and gaskets, Droplet Generation Oil for Probes, and ddPCR Supermix for Probes (no dUTP) (Bio-Rad), and they were performed on the QX200 Droplet Digital PCR System (Bio-Rad), according to the manufacturer’s protocols and established MIQE guidelines for ddPCR [67]. ddPCR assays targeting identified SNP variants were designed by a Bio-Rad’s technician and their sequences are unknown; however, their assay IDs are listed in Additional File 10: Table S2. As a reference, ddPCR assay targeting *β*-actin was used, while for determination of *HTT* transgene copy number, an assay targeting mouse *Rpp30* was used.

### NSC transfection with oligonucleotides

All siRNA oligonucleotides (Additional File 11: Table S3) were synthesized by FutureSynthesis, dissolved in water to 100 μM concentration, and stored at −80 °C. To obtain 20 μM duplexes, sense and antisense strands were diluted in water, heated for 1 min in 90 °C, and kept for gradual cooling at room temperature for 45 min. HD NSCs were then transfected using 2 μL of siPORT Amine (Ambion) and 100 nM oligonucleotides per well of a 6-well plate in 1 mL of complete medium. After 3 h, the medium was replaced with a fresh one, and after the next 24 h, cells were harvested and total RNA was isolated and then used for reverse transcription and ddPCRs, as previously described.

### RNA-Seq

RNA-Seq was performed in the CeNT’s Genomics Core Facility in the Centre of New Technologies, University of Warsaw, Poland. Total RNA isolated from HD iPSCs and NSCs after ribodepletion was sequenced using NovaSeq 6000 system with a pair-end 2x100 cycles procedure. Fastq files were mapped to the reference genome (GENCODE release 42, GRCh38.p13) using STAR (v. 2.7.10b) algorithm. Only the reads mapped to the forward strand were subsetted (samtools 1.16.1). The statistics for reads mapped to the investigated SNPs in *HTT* were retrieved using samtools mpileup (QC parameters: -q 20 -Q 20).

### Statistical analysis

Three biological replicates were included for each experiment. Analyses of obtained results were performed using GraphPad Prism software (version 8.0.1.). Depending on the experimental setup, specific statistical tests were used and are indicated in figure legends. All data are presented as means. Error bars always represent standard deviations.

## Supplementary Information

**Additional file 1: Fig. S1.** Analyses of *ATXN3* and *HTT* assays specificity. Results from ddPCR assays performed on seven samples with predefined ratios of WT/MUT *ATXN3* (A) and *HTT* (B-C) plasmids (I—100% WT and 0% MUT; II—90% WT and 10% MUT; III—75% WT and 25% MUT; IV—50% WT and 50% MUT; V—25% WT and 75% MUT; VI—10% WT and 90% MUT; VII—0% WT and 100% MUT) using ATXN3_SNP5 (A), HTT_SNP5 (B) and HTT_SNP7 assays (C). Mean/Precise values are indicated on WT/MUT bars ± poisson error.

**Additional file 2: Fig. S2.** Immunocytochemistry performed on SCA3 and HD neurons. Exemplary images of immunofluorescent staining of SCA3 neurons for MAP 2 and TUJ1, and HD neurons for DARPP32, TUJ1, MAP 2 and GAD67. DAPI was used for nuclei staining.

**Additional file 3: Fig. S3.** Precise values describing WT/MUT ratio of *ATXN3* in selected SCA3 cells. A) Table with mean %WT and %MUT values obtained using four types of SCA3 cell lines and two SNP assays (mean values from three biological replicates). B-E) Results from ddPCR presented as a mean WT/MUT *ATXN3* transcript allele abundance obtained using ATXN3_SNP2 and ATXN3_SNP5 assays in fibroblasts (B), iPSCs (C), NSCs (D) and neurons (E). To determine statistical significance of differences between WT and MUT allele abundance data were analyzed using unpaired t test. For all experiments presented in this figure *n*=3. Two-tailed p value < 0.05 was considered significant and is depicted in the figure by: *p < 0.05; **p < 0.01; ***p < 0.001; ****p < 0.0001. All data are presented as means ± SD.

**Additional file 4: Fig. S4.** Precise values describing WT/MUT ratio of *HTT* in selected HD cells. A) Table with mean %WT and %MUT values obtained using three types of HD cell lines and three SNP assays (mean values from three biological replicates). B-D) Results from ddPCR presented as a mean WT and MUT *HTT* transcript allele abundance obtained using HTT_SNP2, HTT_SNP5 and HTT_SNP7 assays in iPSCs (B), NSCs (C) and neurons (D). To determine statistical significance of differences between WT and MUT allele abundance, data were analyzed using unpaired t test. For all experiments presented in this figure *n*=3. Two-tailed *p* value < 0.05 was considered significant and is depicted in the figure by: *p < 0.05; **p < 0.01; ***p < 0.001; ****p < 0.0001. All data are presented as means ± SD.

**Additional file 5: Fig. S5.** RNA-Seq analysis of *HTT* reads performed on HD iPSCs and NSCs. A-B) Results from RNA-Seq are presented as a mean WT and MUT *HTT* transcript allele abundance, calculated based on reads from three *HTT* SNP regions (SNP2, SNP5 and SNP7), in iPSCs (A) and NSCs (B). These data were analyzed using unpaired t test. C) Table summarizing precise RNA-Seq results. D-E) Precise results from RNA-Seq presented as a WT and MUT *HTT* transcript allele abundance based on reads from three, particular SNP regions (SNP2, SNP5 and SNP7) in iPSCs (D) and NSCs (E). Data were analyzed using unpaired t test. For all experiments presented in this figure *n*=3. All data are presented as means ± SD. Individual data values are available in Additional File 13.

**Additional file 6: Fig. S6.** Estimation of the total number of endogenous *HTT* transcripts per diploid genome. Results were obtained using *HTT*_SNP7 assay and cDNA from HD iPSCs and NSCs. Data were analyzed using unpaired t test. For all experiments presented in this figure *n*=3. Two-tailed *p* value < 0.05 was considered significant and is depicted in the figure by: *p < 0.05; **p < 0.01; ***p < 0.001; ****p < 0.0001. All data are presented as means ± SD.

**Additional file 7: Fig. S7.** Precise values describing WT/MUT ratio of *HTT* in isogenic controls to HD cells. A) Table with mean %WT and %corrMUT values obtained for lines and presented SNP assays (mean values from three biological replicates). B-C) Results from ddPCR presented as a mean WT/MUT *HTT* transcript allele abundance obtained using HTT_SNP2, HTT_SNP5 and HTT_SNP7 assays in C39 (B) and C105 (C). To determine statistical significance of differences between WT and MUT allele abundance, data were analyzed using unpaired t test. For all experiments presented in this figure *n*=3. Two-tailed *p* value < 0.05 was considered significant and is depicted in the figure by: *p < 0.05; **p < 0.01; ***p < 0.001; ****p < 0.0001. All data are presented as means ± SD. Error bars in the figure represent standard deviations.

**Additional file 8: Fig. S8.** Precise values describing WT/MUT ratio of *HTT* transgene in Hu128/21 mice. A) Table with mean %WT and %MUT values obtained using mice brain tissues and two ddPCR assays (mean values from three biological replicates). B-C) Results from ddPCR presented as a mean WT/MUT *HTT* transcript allele abundance obtained using HTT_SNP2 and HTT_SNP5 assays in 4-month-old (B) and 10-month-old mice (C). Data were analyzed using two-way ANOVA with Tukey’s multiple comparison test. For all experiments presented in this figure *n*=3. Two-tailed *p* value < 0.05 was considered significant and is depicted in the figure by: *p < 0.05; **p < 0.01; ***p < 0.001; ****p < 0.0001. All data are presented as means ± SD. Error bars in the figure represent standard deviations.

**Additional file 9: Table S1.** Table listing all primers and ddPCR assays used in this research with their description.

**Additional file 10: Table S2.** Sequences of oligonucleotides used for silencing of endogenous *HTT* in HD NSCs.

**Additional file 11: Table S3.** A list of primary and secondary antibodies with their dilutions used in immunocytochemistry.

**Additional file 12 **Selected reads from RNA-Seq analysis. Reads covering *HTT* SNPs of interest in each sample (3 samples from HD iPSCs—iH222HD and 3 samples from HD NSCs—nH222HD) selected from aligned bam files (samtools view) and converted to the fastq format (samtools fastq).

**Additional file 13.** Individual data values for selected figures.

## Data Availability

All data generated or analyzed during this study are included in this published article and its supplementary information files. Analyzed RNA-Seq reads are provided as Additional file 12. Individual data values from ddPCR experiments are provided as Additional file 13.

## Abbreviations

PolyQ: Polyglutamine
ddPCR: Droplet digital PCR
WT: Wild type
MUT: Mutant
*HTT*: Huntingtin
*ATXN3*: Ataxin-3
HD: Huntington’s disease
SCA3: Spinocerebellar ataxia type 3
