# Supplementary figures and images for: Allele-specific quantitation of ATXN3 and HTT transcripts in polyQ disease models
Source: BMC Biol. 2023 Feb 1;21:17. doi: 10.1186/s12915-023-01515-3 (PMC9893648; doi:10.1186/s12915-023-01515-3)

## Slide 1
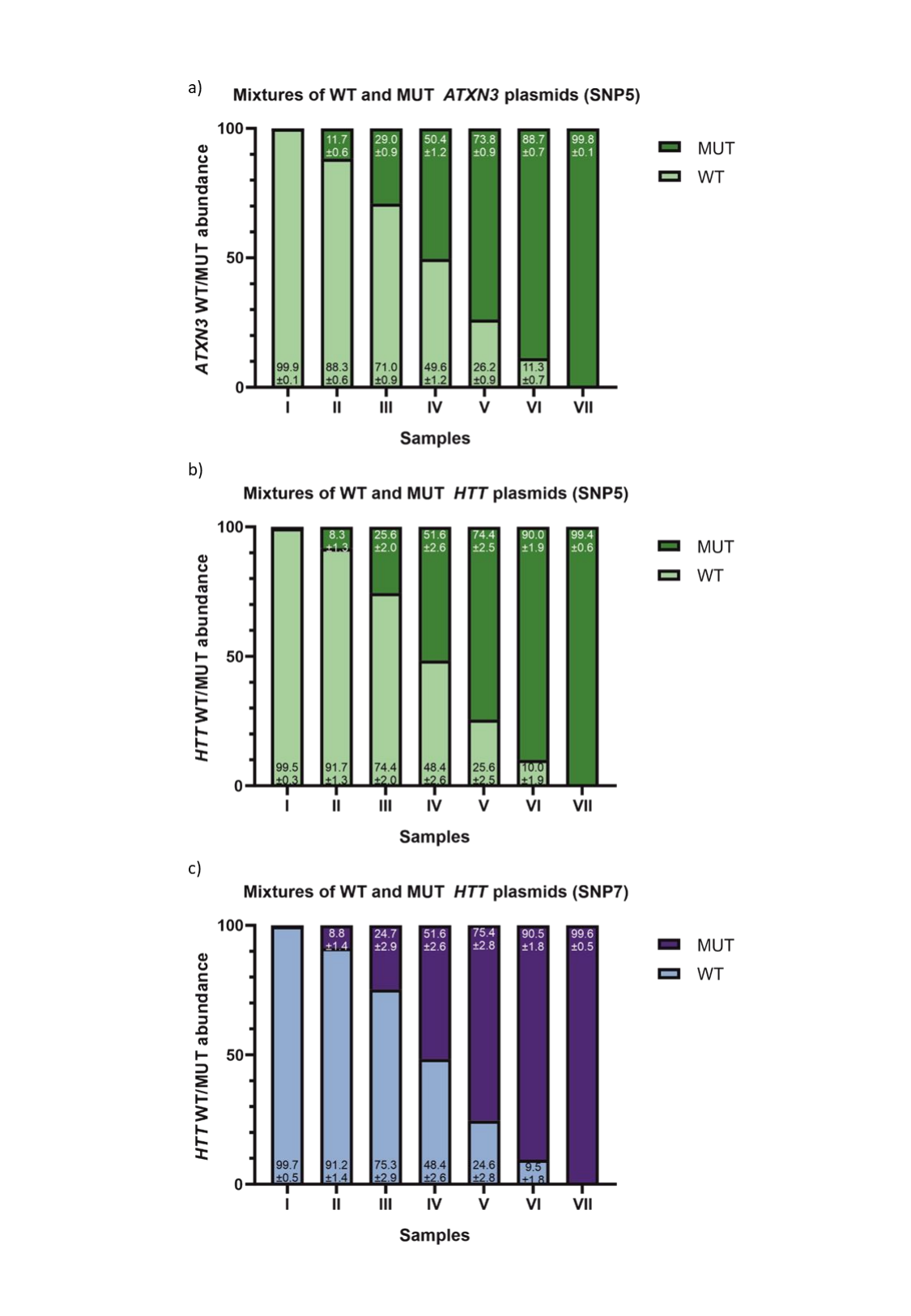

Supplement: Supplementary file 1 — Additional file 1: Fig. S1. Analyses of ATXN3 and HTT assays specificity. Results from ddPCR assays performed on seven samples with predefined ratios of WT/MUT ATXN3 (A) and HTT (B-C) plasmids (I—100% WT and 0% MUT; II—90% WT and 10% MUT; III—75% WT and 25% MUT; IV—50% WT and 50% MUT; V—25% WT and 75% MUT; VI—10% WT and 90% MUT; VII—0% WT and 100% MUT) using ATXN3_SNP5 (A), HTT_SNP5 (B) and HTT_SNP7 assays (C). Mean/Precise values are indicated on WT/MUT bars ± poisson error. [file 12915_2023_1515_MOESM1_ESM.pptx]

## Slide 1
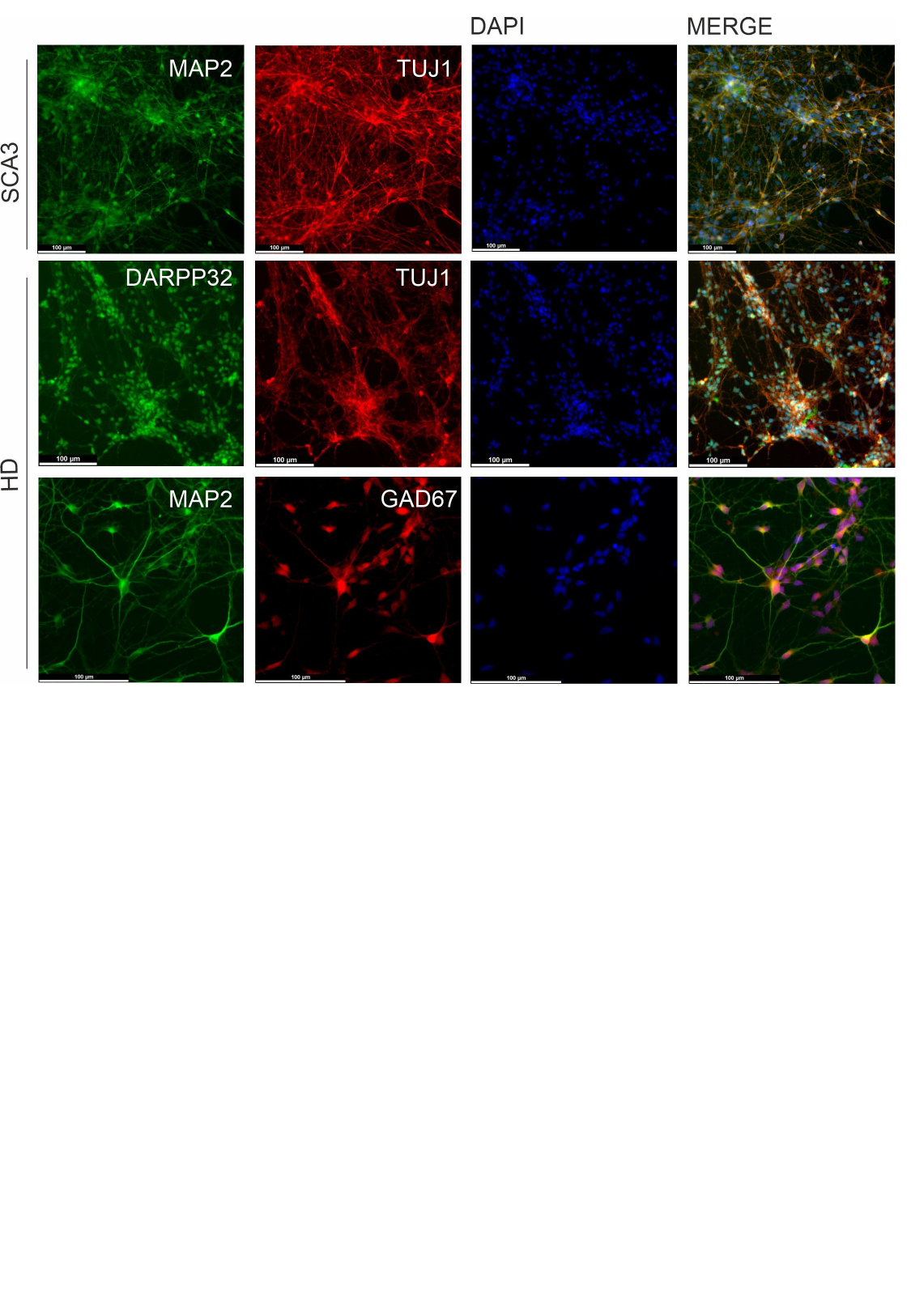

Supplement: Supplementary file 2 — Additional file 2: Fig. S2. Immunocytochemistry performed on SCA3 and HD neurons. Exemplary images of immunofluorescent staining of SCA3 neurons for MAP 2 and TUJ1, and HD neurons for DARPP32, TUJ1, MAP 2 and GAD67. DAPI was used for nuclei staining. [file 12915_2023_1515_MOESM2_ESM.pptx]

## Slide 1
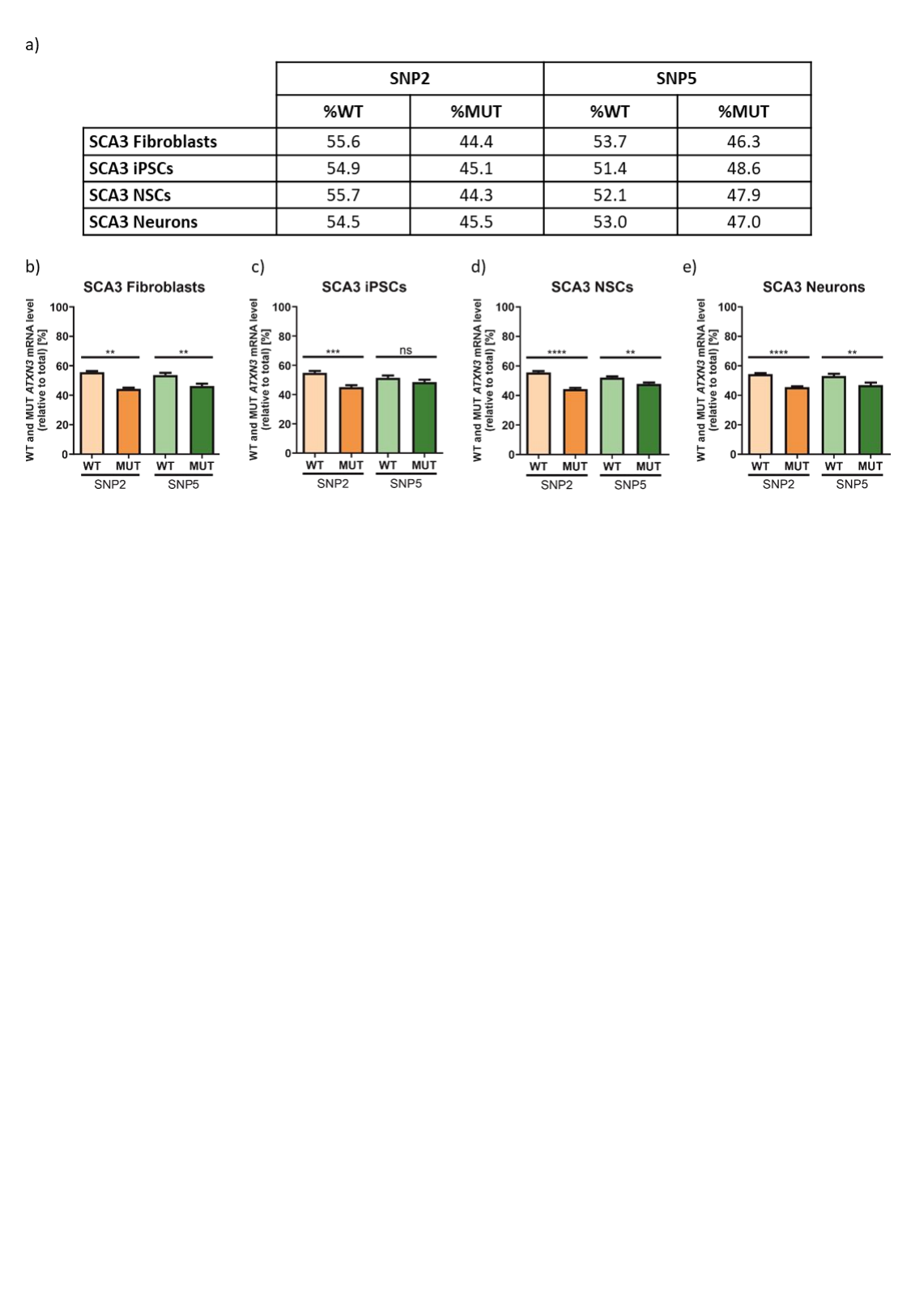

Supplement: Supplementary file 3 — Additional file 3: Fig. S3. Precise values describing WT/MUT ratio of ATXN3 in selected SCA3 cells. A) Table with mean %WT and %MUT values obtained using four types of SCA3 cell lines and two SNP assays (mean values from three biological replicates). B-E) Results from ddPCR presented as a mean WT/MUT ATXN3 transcript allele abundance obtained using ATXN3_SNP2 and ATXN3_SNP5 assays in fibroblasts (B), iPSCs (C), NSCs (D) and neurons (E). To determine statistical significance of differences between WT and MUT allele abundance data were analyzed using unpaired t test. For all experiments presented in this figure n=3. Two-tailed p value < 0.05 was considered significant and is depicted in the figure by: *p < 0.05; **p < 0.01; ***p < 0.001; ****p < 0.0001. All data are presented as means ± SD. [file 12915_2023_1515_MOESM3_ESM.pptx]

## Slide 1
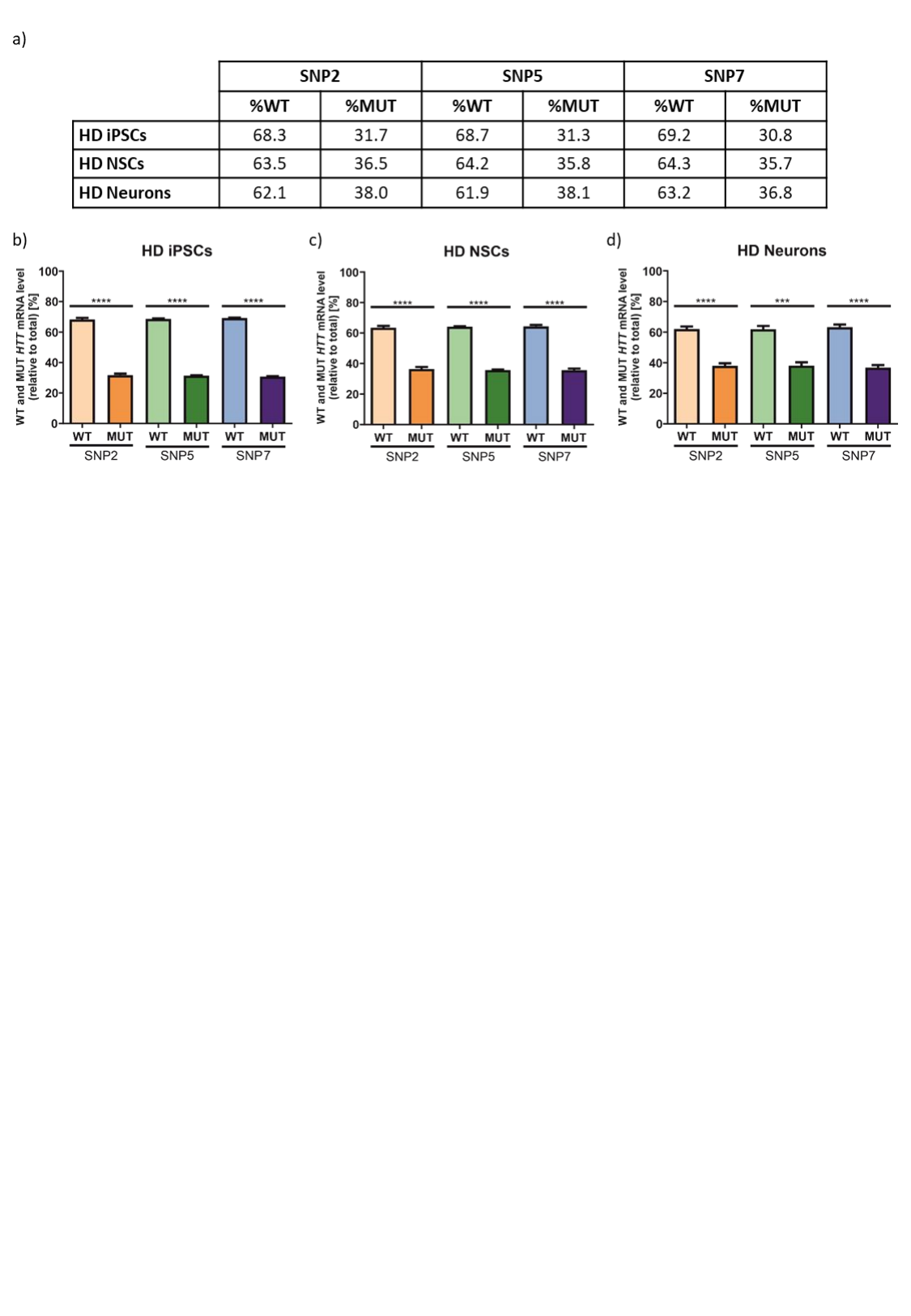

Supplement: Supplementary file 4 — Additional file 4: Fig. S4. Precise values describing WT/MUT ratio of HTT in selected HD cells. A) Table with mean %WT and %MUT values obtained using three types of HD cell lines and three SNP assays (mean values from three biological replicates). B-D) Results from ddPCR presented as a mean WT and MUT HTT transcript allele abundance obtained using HTT_SNP2, HTT_SNP5 and HTT_SNP7 assays in iPSCs (B), NSCs (C) and neurons (D). To determine statistical significance of differences between WT and MUT allele abundance, data were analyzed using unpaired t test. For all experiments presented in this figure n=3. Two-tailed p value < 0.05 was considered significant and is depicted in the figure by: *p < 0.05; **p < 0.01; ***p < 0.001; ****p < 0.0001. All data are presented as means ± SD. [file 12915_2023_1515_MOESM4_ESM.pptx]

## Slide 1
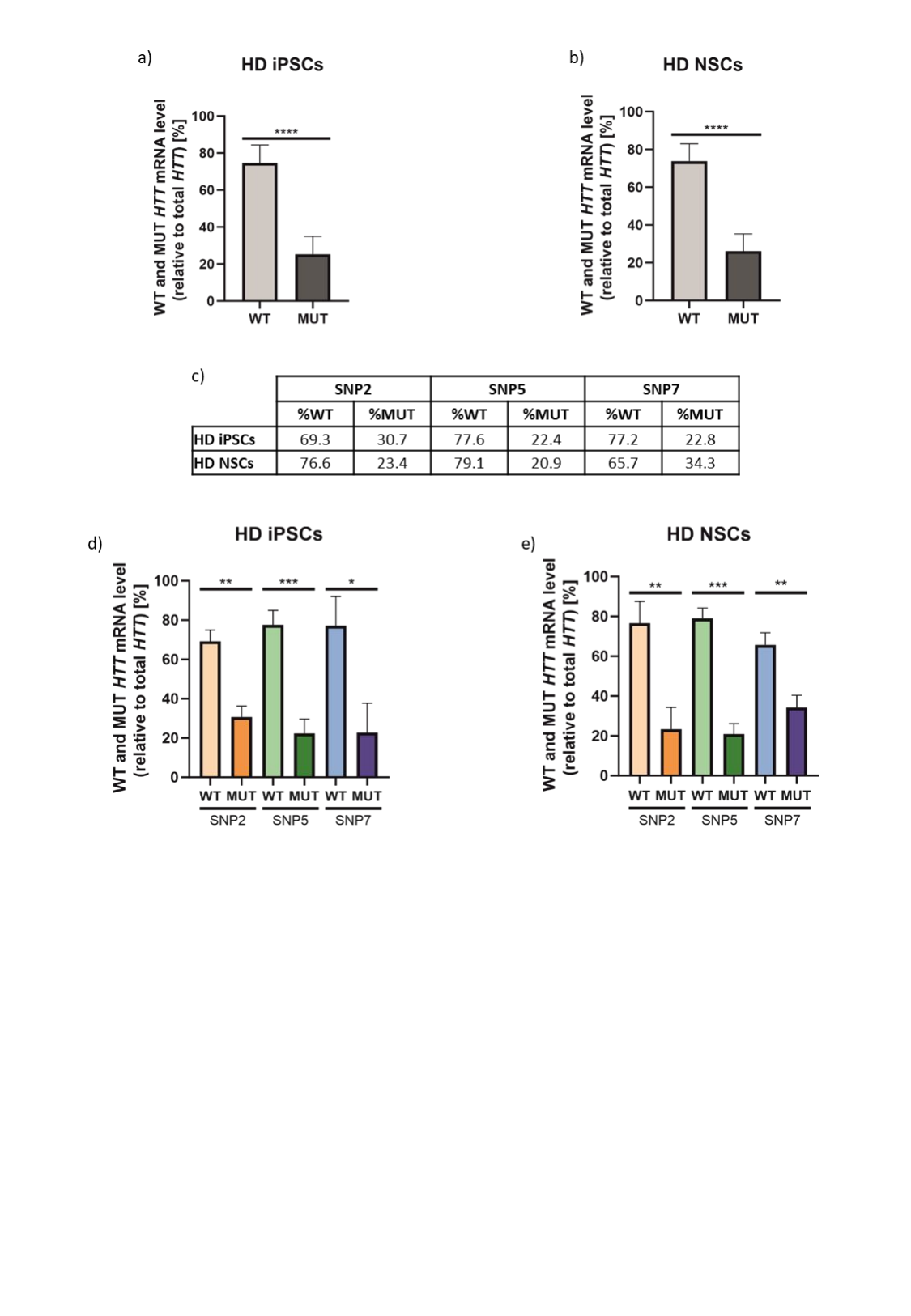

Supplement: Supplementary file 5 — Additional file 5: Fig. S5. RNA-Seq analysis of HTT reads performed on HD iPSCs and NSCs. A-B) Results from RNA-Seq are presented as a mean WT and MUT HTT transcript allele abundance, calculated based on reads from three HTT SNP regions (SNP2, SNP5 and SNP7), in iPSCs (A) and NSCs (B). These data were analyzed using unpaired t test. C) Table summarizing precise RNA-Seq results. D-E) Precise results from RNA-Seq presented as a WT and MUT HTT transcript allele abundance based on reads from three, particular SNP regions (SNP2, SNP5 and SNP7) in iPSCs (D) and NSCs (E). Data were analyzed using unpaired t test. For all experiments presented in this figure n=3. All data are presented as means ± SD. Individual data values are available in Additional File 13. [file 12915_2023_1515_MOESM5_ESM.pptx]

## Slide 1
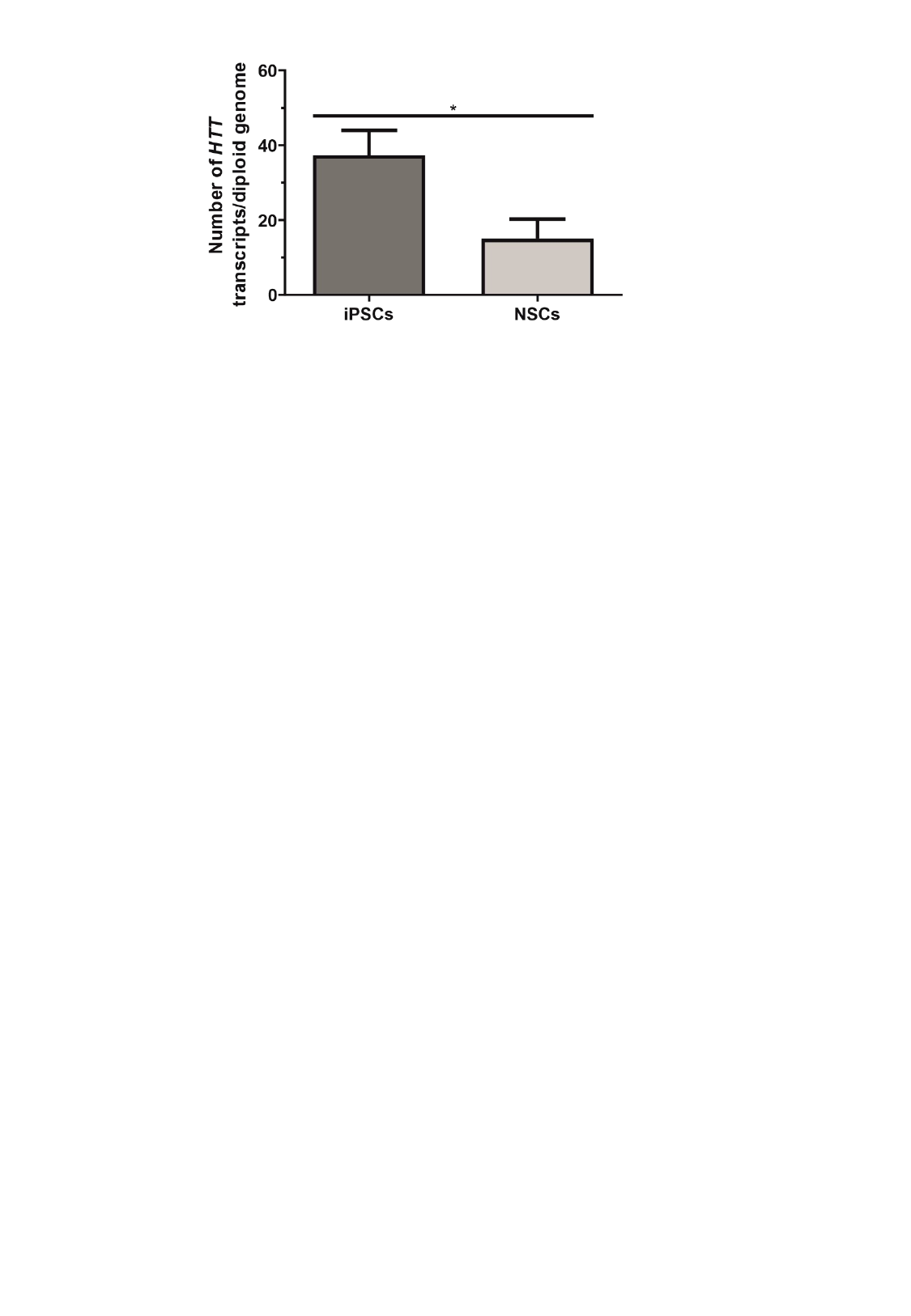

Supplement: Supplementary file 6 — Additional file 6: Fig. S6. Estimation of the total number of endogenous HTT transcripts per diploid genome. Results were obtained using HTT_SNP7 assay and cDNA from HD iPSCs and NSCs. Data were analyzed using unpaired t test. For all experiments presented in this figure n=3. Two-tailed p value < 0.05 was considered significant and is depicted in the figure by: *p < 0.05; **p < 0.01; ***p < 0.001; ****p < 0.0001. All data are presented as means ± SD. [file 12915_2023_1515_MOESM6_ESM.pptx]

## Slide 1
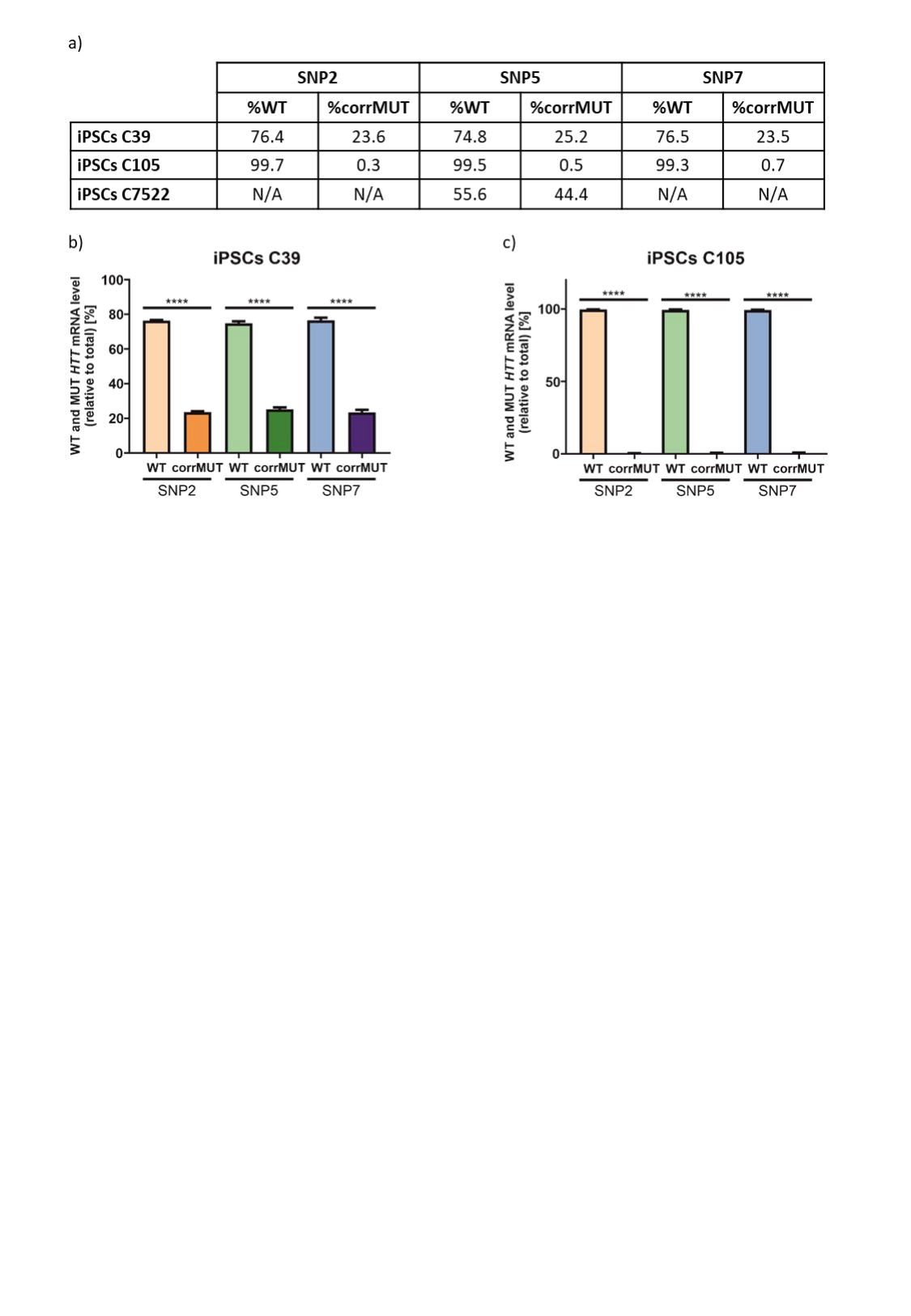

Supplement: Supplementary file 7 — Additional file 7: Fig. S7. Precise values describing WT/MUT ratio of HTT in isogenic controls to HD cells. A) Table with mean %WT and %corrMUT values obtained for lines and presented SNP assays (mean values from three biological replicates). B-C) Results from ddPCR presented as a mean WT/MUT HTT transcript allele abundance obtained using HTT_SNP2, HTT_SNP5 and HTT_SNP7 assays in C39 (B) and C105 (C). To determine statistical significance of differences between WT and MUT allele abundance, data were analyzed using unpaired t test. For all experiments presented in this figure n=3. Two-tailed p value < 0.05 was considered significant and is depicted in the figure by: *p < 0.05; **p < 0.01; ***p < 0.001; ****p < 0.0001. All data are presented as means ± SD. Error bars in the figure represent standard deviations. [file 12915_2023_1515_MOESM7_ESM.pptx]

## Slide 1
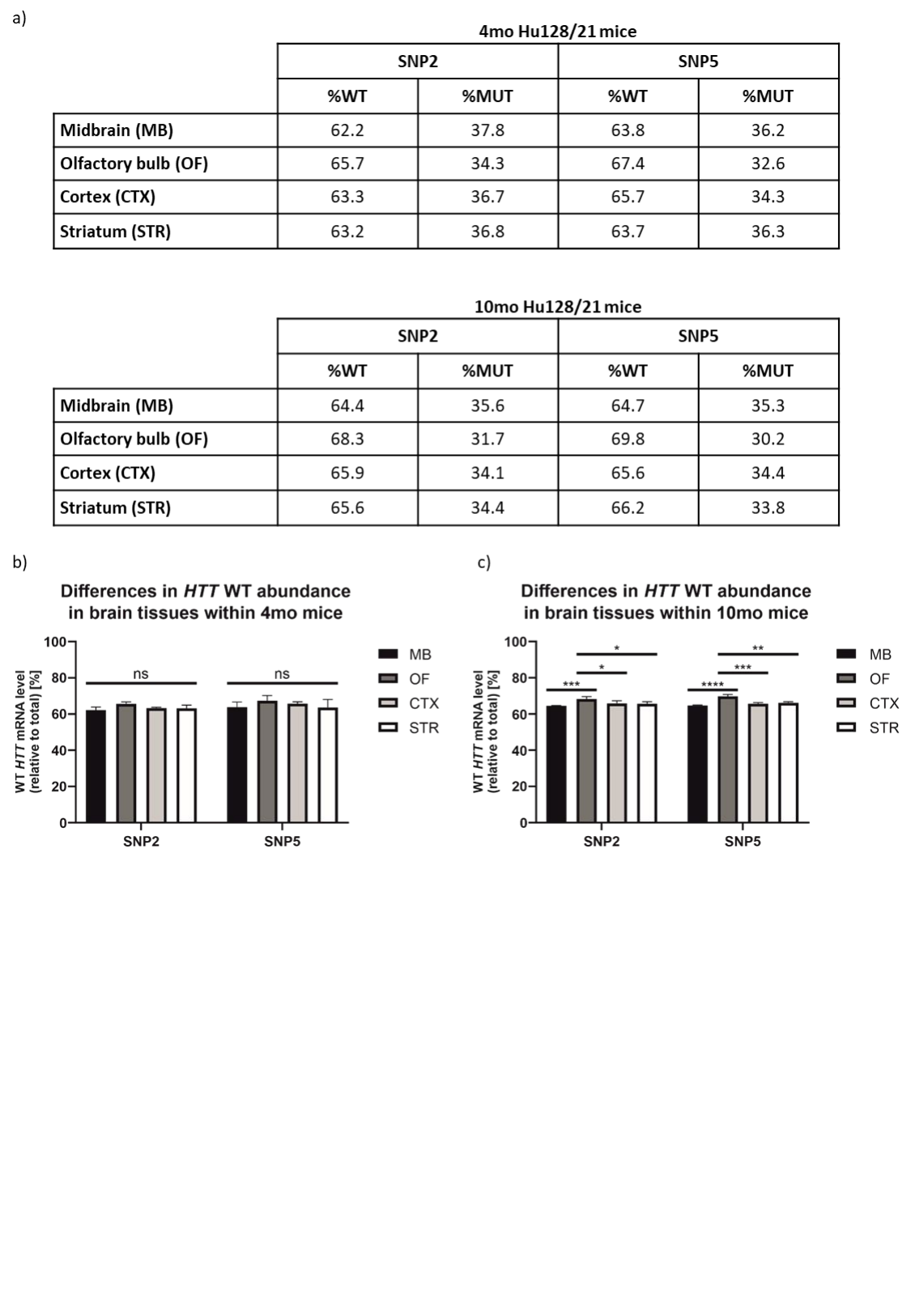

Supplement: Supplementary file 8 — Additional file 8: Fig. S8. Precise values describing WT/MUT ratio of HTT transgene in Hu128/21 mice. A) Table with mean %WT and %MUT values obtained using mice brain tissues and two ddPCR assays (mean values from three biological replicates). B-C) Results from ddPCR presented as a mean WT/MUT HTT transcript allele abundance obtained using HTT_SNP2 and HTT_SNP5 assays in 4-month-old (B) and 10-month-old mice (C). Data were analyzed using two-way ANOVA with Tukey’s multiple comparison test. For all experiments presented in this figure n=3. Two-tailed p value < 0.05 was considered significant and is depicted in the figure by: *p < 0.05; **p < 0.01; ***p < 0.001; ****p < 0.0001. All data are presented as means ± SD. Error bars in the figure represent standard deviations. [file 12915_2023_1515_MOESM8_ESM.pptx]
