# Supplementary material for: Allele-specific quantitation of ATXN3 and HTT transcripts in polyQ disease models
Source: BMC Biol. 2023 Feb 1;21:17. doi: 10.1186/s12915-023-01515-3 (PMC9893648; doi:10.1186/s12915-023-01515-3)
Supplement: Supplementary file 9 — Additional file 9: Table S1. Table listing all primers and ddPCR assays used in this research with their description. [file 12915_2023_1515_MOESM9_ESM.docx]

| **Primary antibodies** | **Dilution** | **Manufacturer** |
| --- | --- | --- |
| DARPP-32 (19A3) | 1:400 | Cell Signaling Technology (Cat. No. 2306) |
| GAD67 | 1:50 | Santa Cruz Biotechnology (Cat. No. SC-28376) |
| MAP2 | 1:200 | Cell Signaling Technology (Cat. No. 4542) |
| TUJ1 | 1:500 | BioLegend (Cat. No. MMS-435P) |
| **Secondary antibodies** | **Dilution** | **Manufacturer** |
| anti-rabbit Alexa Fluor 488 | 1:1000 | **Jackson ImmunoResearch** (711-546-152) |
| anti-mouse Alexa Fluor 594 | 1:1000 | **Jackson ImmunoResearch** (715-586-151) |

**Supplementary Table 1.** A list of primary and secondary antibodies with their dilutions used in immunocytochemistry.
