# Supplementary material for: Allele-specific quantitation of ATXN3 and HTT transcripts in polyQ disease models
Source: BMC Biol. 2023 Feb 1;21:17. doi: 10.1186/s12915-023-01515-3 (PMC9893648; doi:10.1186/s12915-023-01515-3)
Supplement: Supplementary file 10 — Additional file 10: Table S2. Sequences of oligonucleotides used for silencing of endogenous HTT in HD NSCs. [file 12915_2023_1515_MOESM10_ESM.docx]

| **Target Gene** | **Primer sequence (5' -> 3')** | **Description** |
| --- | --- | --- |
| **ATXN3_Fwd** | GGCCGTTGGCTCCAGAC | Cloning for SNP identification |
| **ATXN3_Rev** | AGATCCACTAAGTACTGTGACTTC | Cloning for SNP identification |
| **HTT_Fwd** | TTCTGCTTTTACCTGCGGCC | Cloning for SNP identification |
| **HTT_Rev** | AGTGTTCCCAAAGCCTGCTCAC | Cloning for SNP identification |
| **HTT_RT** | CTGTCTCAGAGCTGCTGACATAAC | RT for SNP identification |
| **ATXN3_SNP2** |  | ddPCR assay ID: dMDS306026999 |
| **ATXN3_SNP5** |  | ddPCR assay ID: dHsaMDS942717091 |
| **HTT_SNP2** |  | ddPCR assay ID: dMDS291328664 |
| **HTT_SNP5** |  | ddPCR assay ID: dMDS735189637 |
| **HTT_SNP7** |  | ddPCR assay ID: dMDS165698820 |
| **ACTB** |  | ddPCR assay ID: dHsaCPE5190199 |
| **Rpp30** |  | ddPCR assay ID: dMmuCNS822293939 |

**Supplementary Table 2.** Table listing all primers and ddPCR assays used in this research with their description.
