# Supplementary material for: Allele-specific quantitation of ATXN3 and HTT transcripts in polyQ disease models
Source: BMC Biol. 2023 Feb 1;21:17. doi: 10.1186/s12915-023-01515-3 (PMC9893648; doi:10.1186/s12915-023-01515-3)
Supplement: Supplementary file 11 — Additional file 11: Table S3. A list of primary and secondary antibodies with their dilutions used in immunocytochemistry. [file 12915_2023_1515_MOESM11_ESM.docx]

| **Name** | **Sense (5'-3')** | **Antisense (5'-3')** |
| --- | --- | --- |
| A2 | GCUGCUGC**A**GCUGCUGCUGCU | GCUGCUGC**A**GCUGCUGCUGCU |
| siHTT | GCCUUCGAGUCCCUCAAGUCC | ACUUGAGGGACUCGAAGGCCU |
| siRluc | AUCUGAAGAAGGAGAAAAATT | AUCUGAAGAAGGAGAAAAATT |

**Supplementary Table 3.** Sequences of oligonucleotides used for silencing of endogenous HTT in HD NSCs.
